# The impact of hypoxia preconditioning on mesenchymal stem cells performance in hypertensive kidney disease

**DOI:** 10.1186/s13287-024-03778-1

**Published:** 2024-06-09

**Authors:** Gurparneet Kaur Sohi, Naba Farooqui, Arjunmohan Mohan, Kamalnath Sankaran Rajagopalan, Li Xing, Xiang Y. Zhu, Kyra Jordan, James D. Krier, Ishran M. Saadiq, Hui Tang, LaTonya J. Hickson, Alfonso Eirin, Lilach O. Lerman, Sandra M. Herrmann

**Affiliations:** 1 Division of Nephrology and Hypertension, Mayo Clinic, 200, First Street SW, Rochester, 55902 MN USA; 2 Division of Nephrology and Hypertension, Mayo Clinic, Jacksonville, FL USA; 3 Department of Urology, Zhongda Hospital, Southeast University, Nanjing, Jiangsu province China

**Keywords:** Hypertensive kidney disease, Adipose mesenchymal stem cells, Hypoxia preconditioning, Angiogenesis, Senescence

## Abstract

**Background:**

Autologous mesenchymal stem cells (MSCs) have emerged as a therapeutic option for many diseases. Hypertensive kidney disease (HKD) might impair MSCs’ reparative ability by altering the biomolecular properties, but the characteristics of this impairment are unclear. In our previous pre-clinical studies, we found hypoxic preconditioning (HPC) enhanced angiogenesis and suppressed senescence gene expression. Thus, we hypothesize that HPC would improve human MSCs by enhancing their functionality and angiogenesis, creating an anti-inflammatory and anti-senescence environment.

**Methods:**

MSC samples (*n* = 12 each) were collected from the abdominal fat of healthy kidney donors (HC), hypertensive patients (HTN), and patients with hypertensive kidney disease (HKD). MSCs were harvested and cultured in Normoxic (20% O_2_) or Hypoxic (1% O_2_) conditions. MSC functionality was measured by proliferation assays and cytokine released in conditioned media. Senescence was evaluated by senescence-associated beta-galactosidase (SA-beta-gal) activity. Additionally, transcriptome analysis using RNA-sequencing and quantitative PCR (qPCR) were performed.

**Results:**

At baseline, normoxic HTN-MSCs had higher proliferation capacity compared to HC. However, HPC augmented proliferation in HC. HPC did not affect the release of pro-angiogenic protein VEGF, but increased EGF in HC-MSC, and decreased HGF in HC and HKD MSCs. Under HPC, SA-β-gal activity tended to decrease, particularly in HC group. HPC upregulated mostly the pro-angiogenic and inflammatory genes in HC and HKD and a few senescence genes in HKD.

**Conclusions:**

HPC has a more favorable functional effect on HC- than on HKD-MSC, reflected in increased proliferation and EGF release, and modest decrease in senescence, whereas it has little effect on HTN or HKD MSCs.

**Supplementary Information:**

The online version contains supplementary material available at 10.1186/s13287-024-03778-1.

## Significance statement

In this study, we evaluate the effect of hypoxic preconditioning on human mesenchymal stem cells characteristics from healthy individuals and patients with hypertension with and without kidney disease providing adaptative mechanisms involving molecular and functional alterations when compared to normoxic conditions. The differential gene expression patterns highlight the tissue repair which is unique to each group. This research enhances our understanding of cellular and molecular dynamics in diseased states, and the effects of hypoxic preconditioning on MSC performance.

**Supplementary Information:**

The online version contains supplementary material available at 10.1186/s13287-024-03778-1.

## Introduction

Chronic kidney disease (CKD) is an escalating worldwide public health problem and has become more prevalent in the American population. CKD affects more than 1 in 7 adults in the United States (US) which is 15% of US adults [1]. As the disease progresses patients with CKD may eventually require dialysis or kidney transplantation. Hypertension (HTN) is a chronic state of uncontrolled high blood pressure leading to hypertensive nephropathy, which damages the small blood vessels in the kidneys, resulting in reduced glomerular filtration rate (GFR) [2]. HTN is a cardinal risk factor for hypertensive kidney disease (HKD) and a major cause of end-stage kidney disease (ESKD) [3]. HKD is characterized by remodeling and loss of renal microvasculature that turns into irreversible tissue injury with progressive renal function decline [4]. The renal arterioles undergo a subset of changes such as medial hypertrophy and intimal fibrosis. This subsequently causes endothelial damage leading to hyaline arteriolosclerosis which further decreases renal blood flow causing glomerular ischemia and in turn leads to glomerular and tubular fibrosis along with atrophy ultimately resulting in glomerulosclerosis [5]. These damaging events ignite localized hypoxia and fibrosis resulting in the deterioration of kidney function.

Renal stem cells keep regenerating and repairing the nephrons throughout life but due to the decline in glomerular functions, the damaged nephrons have limited regenerative capacity [6]. Mesenchymal stem cells (MSC) are situated throughout the body as pericytes cells embedded within the vascular basement of blood microvessels mediating physiological and pathological repair processes shaping the structure of blood vessels [7, 8]. Impairment of the molecular and cellular processes due to oxidative stress and DNA damage, blunts reparative functions of stem cells, further contributing to chronic glomerulosclerosis and tubulointerstitial fibrosis [9, 10]. Advances in regenerative cell therapies have sparked interest in the use of MSC due to their immunomodulatory, anti-inflammatory, pro-angiogenetic, and pro-regenerative properties. The paracrine signaling of MSCs drives the renoprotective functions facilitating renal repair [11]. Despite significant understanding, these properties in an older population with HKD are poorly appreciated. As MSCs have been shown to be promising regenerative therapies due to their multipotency and availability they could be used as a novel therapy capable of delaying HKD progression. Hypoxic preconditioning (HPC) further enhances this regenerative capacity, emerging as a pivotal strategy in regenerative medicine, particularly in optimizing the therapeutic effectiveness of MSCs. This combination highlights the potential synergy between the inherent properties of MSCs and the strategic application of HPC in the context of therapeutic interventions for HKD. In our previous in vitro pre-clinical studies using a swine hypertensive model of kidney disease, HPC has been shown to enhance angiogenesis and improve migratory and proliferative capacity mitigating MSC dysfunction and senescence of MSCs from pigs with atherosclerotic renovascular hypertensive kidney disease (ARAS) [12]. While in vivo renal artery injection of HPC-treated MSCs was efficacious in reducing kidney tissue fibrosis, and inflammation in addition to reducing diastolic blood pressure in pigs with ARAS [13].

In this study, we compared the MSC functionality, secretome, and transcriptome profile among participants of HKD, hypertension without CKD, and healthy control groups and evaluated the effects of HPC within the three groups. We hypothesize that HPC would improve human MSCs’ by enhancing their functionality and angiogenesis, creating an anti-inflammatory and anti-senescence environment.

## Methods and materials

### Study participants

Adipose tissue-derived MSCs were obtained from healthy control kidney donors (HC), patients with hypertension but without kidney disease (HTN), and patients with HKD (*n* = 12 each group). Patients with ages between 18 and 80 years, with estimated glomerular filtration rate (eGFR) ≥ 15 mL/min/1.73m^2^ were screened and recruited at Mayo Clinic (Rochester, MN). A written informed consent was obtained from the subjects for undergoing abdominal fat biopsy. There were no restrictions on anti-hypertensives or anti-lipid drugs before the study. Exclusion criteria for the study were: diabetes mellitus, pregnancy, evidence of hepatitis B or C, or HIV infection, history of solid organ transplantation, nephrotic syndrome, immunosuppression therapy, active glomerulonephritis, autosomal dominant or recessive polycystic kidney disease, history of reno-vascular disease (RVD), active malignancy, active substance abuse or history of substance abuse (including alcohol and smoking) within the past 2 years, body weight > 150 kg or BMI > 50 Kg/m^2^, uncontrolled psychiatric disorder.

The patient population who fulfilled the criteria for the study were selected as : (1) healthy volunteers having no history of HTN or HKD with (eGFR > 80 ml/min/1.73m^2^ by iothalamate clearance measured GFR) without microalbuminuria; (2) Non-diabetic patients with eGFR > 60 ml/min/1.73m^2^, without microalbuminuria, diagnosed with blood pressure > 140/90mmHg or with a history of hypertension controlled by antihypertensive drugs (HTN); (3) Patients clinically diagnosed as HKD (eGFR < 60 mL/min/1.73m^2^) with non-diabetic or other overt etiologies of kidney diseases, with blood pressure > 140/90mmHg or with antihypertensives controlled hypertension.

### MSC isolation and characterization

MSCs were isolated from the abdominal adipose tissue of each patient in an outpatient surgery suite under sterile conditions. They were cultured and expanded as described in our previous studies [14–16]. After harvesting, the adipose tissue was processed under sterile conditions by mincing using a gentleMACS Octo tissue dissociator and digested in collagenase-H at 37 °C for 45 min. Media containing serum was added to the enzymatically digested suspension to neutralize the reaction. The suspension was filtered through a 100 μm cell strainer to remove the residual tissue and then centrifuged to form a pellet of cells. Cells were resuspended in Advanced Minimum Essential Medium (Gibco™, ThermoFisher, Cat. # 12,492,013) supplemented with 5% platelet lysate (PLTmax, Mill Creek Life Sciences, Rochester, MN, USA), 5 ml of GlutaMAX Supplement (Gibco™, ThermoFisher, Cat. # 35,050,061), Broad Spectrum Antibiotics, 1 ml of Heparin and expanded in culture for 3–5 passages under sterile conditions at 37 °C with 5% CO². Non-adherent cells were removed by replacing the culture medium every 2 days. When MSCs achieved 80% confluency, adherent cells were treated with Trypsin (TrypLE™ Express Gibco BRL, Waltham, MA) and subcultured. MSCs were characterized according to the International Society for Cellular Therapy (ISCT) by imaging flow cytometry (FlowSight, Amnis, Seattle, WA, USA) to confirm the expression of MSC-specific surface markers CD73 (R&D Systems®, Minneapolis, MN, USA, Cat. # AF4488), CD90 (Abcam, San Francisco, CA, USA Cat. # ab124527), and CD105 (Abcam, Cat. # ab53321). Same way, negative cell surface markers for MSCs CD45 (Abcam, Cat. # ab51482), CD34 (BD Biosciences, San Jose, CA, Cat. # 340,441), or CD14 (Abcam, Cat. # ab82012) were also imaged using flow cytometry [17–22]. The cell surface markers were used according to the protocol recommended by the manufacturer. Briefly, single cells were visualized using the aspect ratio intensity and area of brightfield on the FlowSight. Determination of thresholds is assessed by observing histograms of single-stained controls (AbC™ Total Antibody Compensation Bead Kit, Molecular Probes, Eugene, OR, USA, Cat. # A10497). The appropriate gate was established and adjusted according to positively stained single cells in the scatterplot. Images were analyzed using Amnis® Image Data Exploration and Analysis Software (IDEA version 11.2).

### MSCs transdifferentiation

MSCs between passages 3–5 were cultured to assess their tri-lineage differentiation into adipocytes, chondrocytes, and osteocytes. This was achieved following the manufacturer’s instructions for a commercial kit (R & D Systems, MN, Catalog # SC006). In all cultures, the medium was replaced every 2–4 days, and cells were studied at 21 days [23]. 

### Adipogenic differentiation

MSCs (2.1 × 10^4^/cm^2^) were cultured with StemXVivo adipogenic medium (R&D Systems) containing hydrocortisone, isobutylmethylxanthine, and indomethacin using a 24-well plate. Cells were cultured to 100% confluence, and at 21 days adipocytes were set for detection of fatty acid binding protein 4.

### Osteogenic differentiation

MSCs (4.2 × 10^3^/cm^2^) were cultured with StemXVivo Osteogenic medium (R&D Systems) containing dexamethasone, ascorbate-phosphate, and β-glycerolphosphate using a 24-well plate. Cells were cultured to 50–70% confluence, and at 21 days osteocytes were set for detection of osteocalcin.

### Chondrogenic differentiation

MSCs (2.5 × 10^5^) after transferring to a 15-ml conical tube were centrifuged, and cultured in a chondrogenic differentiation medium containing insulin, transferrin, selenious acid, linoleic acid, dexamethasone, ascorbate-phosphate, proline and pyruvate. At 21 days the chondrogenic pellet was yielded for detection of aggrecan.

### Hypoxia preconditioning protocol

MSCs were cultured and maintained between passages 3–5 under normoxic conditions (with 20% O_2_ at 37 °C) or hypoxic conditions (with 1% O_2_ at 37 °C) for 48–72 h. MSCs were placed in a Modular Incubator Chamber (Billumps-Rothenberg; Del Mar, CA, USA) that was flushed with a mixture of 1% O2, 5% CO2, and 94% N2 to accomplish hypoxic conditions and confirmed by an infrared gas analyzer (Novametrics, Wallingford, CT, USA) [12].

### MSC proliferation functional studies

The proliferation of hypoxic- and normoxic- MSCs was assessed by calorimetric staining after incubating cells in 96-well plates in respective chambers for 24 h, followed by 4 h of incubation with MTS/PMS solution (CellTiter 96® AQueous Non-Radioactive Cell Proliferation Assay, Promega, Madison, WI, USA, Cat. #G5421) [12, 24].

### MSC secretome functional studies

MSC secretome function was assessed by measuring soluble angiogenetic and inflammatory factors released in conditioned culture media. MSCs were cultured in 10 cm Petri dishes containing serum-free conditioned media and incubated in normoxic and hypoxic conditions for 24 h. Media supernatants of respective cells were collected and stored at − 80 °C until assayed. Angiogenic, Senescence and Inflammatory factors were measured using MILLIPLEX® Human Cytokine/Chemokine Magnetic Bead Panel Immunology Multiplex Assay for Vascular Endothelial Growth Factor (VEGF), Interleukin 1-alpha (IL-1α), Interferon-gamma (IFN-γ), Tumor Necrosis Factor-alpha (TNF-α), Interleukin 6 (IL-6), Monocyte Chemoattractant Protein-1 (MCP-1), Interleukin-8 (IL-8), Epidermal Growth Factor (EGF). Similarly, Human Growth Factor (HGF; Sigma-Aldrich, St. Louis, MO, USA, Cat. # RAB1135) and Epidermal Growth Factor (EGF; MyBioSource, San Diego, CA, USA, Cat. # DEGOO) by enzyme-linked immunosorbent assays (ELISAs) according to manufacturer protocols and the corrected readings were standardized according to the live cell counts of the respective sample.

### MSC senescence

Senescence of Hypoxic and Normoxic MSCs was evaluated by quantitative PCR assay with IDs P16 (Hs00923894), P21 (Hs00355782), and by senescence-associated β-galactosidase (SA-β-gal) activity using a Cellular Senescence Activity Assay kit v(Enzo Life Sciences Inc., Farmingdale, NY, USA, Cat. #ENZ-KIT129-0120) performed according to manufacturer’s instruction [12].

### Quantitative PCR (qPCR)

Fold change of gene expression was obtained using ΔΔCT method on RNA isolation, cDNA synthesis, and quantitative-PCR was performed using TaqMan™ on Hypoxic and Normoxic MSCs as described previously [12]. Candidates for the Assay IDs are as follows: *EGF* (Hs0199990), *VEGF* (Hs00900055), P16 (Hs00923894), P21 (Hs00355782), and *GAPDH* (Hs02786624) was used as a reference control for both Hypoxic and Normoxic MSCs.

### RNA-sequencing (seq) analysis in MSCs

The raw RNA sequencing paired-end reads for the samples were processed through the Mayo RNA-Seq bioinformatics pipeline, MAP-RSeq version 3.1.4 [25], which uses a very fast, accurate, and splice-aware aligner, STAR [26] to align reads to the reference human genome build hg38. Gene and exon expression quantification was performed using the Subread [27] package to obtain both raw and normalized (FPKM – Fragments Per Kilobase per Million mapped reads) reads. Using the raw gene counts report from MAP-RSeq, differentially expressed genes were identified using the bioinformatics package edgeR 2.6.2 [28]. Heat maps and volcano plots were generated using Morpheus (https://software.broadinstitute.org/morpheus/) and RStudio (package–enhanced volcano), respectively from the list of genes on the MGI database (http://www.informatics.jax.org/genes.shtml) filtered based on the expression of Angiogenesis, Inflammation, and Senescence in both Hypoxic and Normoxic MSCs.

The mRNA sequencing was done as described in our previous studies [29, 30], and the gene expression was standardized using several reads/samples for gene length by FPKM (fragments/kilobase pair/million mapped reads). Genes with RPKM > 0.1 with a fold change ≥ 1.5 and a *p*-value ≤ 0.05 were considered to be upregulated in HC/HTN/HKD MSCs, and similar genes with a fold change ≤ -1.5 and a *p*-value ≤ 0.05 were considered to be downregulated. Bioinformatic analysis was performed using Metascape (http://metascape.org) and differentially expressed genes were categorized based on their biological process.

### Statistical analysis

All continuous variables were expressed as mean ± standard deviation (SD) and analyzed using JMP 17.0.0 software and BlueSky Statistics. The graphs were generated using GraphPad Prism v9.0.2. The normality assumption was tested using the Shapiro-Wilk Test. Two-sample or Wilcoxon tests were used for comparisons between groups as appropriate. Differences between the genetic markers of HC, HTN, and HKD, as well as clinical characteristics of patient groups in the study, were tested using the two-sample or Wilcoxon tests. Correlation analysis was used to test associations between the patient’s age and MSC proliferation during normoxia using BlueSky Statistics (BlueSky Statistics LLC, Chicago, IL, USA) version 7.40. A *p*-value of < 0.05 was considered statistically significant. Search Tool for the Retrieval of Interacting Genes (STRING) version 12.0 (http://string-db.org/) was used to predict associations among differentially expressed genes and transcription factors of MSCs of HC, HTN, HKD groups.

## Results

The baseline characteristics of HC, HTN, and HKD patients used in the study are summarized in Table 1. HKD group patients were older than HC and HTN groups. Blood pressure was higher in HTN and HKD as compared to the HC group, despite being on anti-hypertensive medications. Under statin therapy, HTN and HKD groups had lower Low-Density Lipid Lipoprotein (LDL) levels than the HC group. Kidney function was lower in HKD in comparison to the HC and HTN groups.

**Table 1:** Baseline demographics

VARIABLES	HC (12)	HTN (12)	HKD (12)	*P* value
Age (Years) (IQR)	52.5 (47-63.5)	57.5 (36-71.5)	72 (71.2–74.7) ^*#^	0.035
Gender (female/male)	6/6	6/6	6/6	NS
BMI (Kg/m^2^) (IQR)	27.21 (26.42–29.9)	28.7 (24.4–33.2)	26.55 (24.09–31.6)	NS
SBP (mmHg) (IQR)	114.5 (108-120.5)	129.5 (121.2-136.2) ^*^	127 (117.2-142.7) ^*^	0.03
DBP (mmHg) (IQR)	67 (64–75)	78.5 (70.7–90.2) ^*^	76 (72.5–78.5)	0.03
Serum Glucose (mg/dL) (IQR)	96.5(93-102.2)	97 (91.5-101.5)	96 (92–108)	NS
Serum Creatinine (mg/dL) (IQR)	0.9 (0.8–0.9)	0.94 (0.8–1.1)	1.93 (1.48–2.21) ^*#^	< 0.001
eGFR (CKD-EPI mL/min)	86.5 (79.7–90)	83 (62.5–88.5)	34 (26-40.7) ^*#^	< 0.001
Urine spot Protein/Osmolality	0.19 (0.1–0.2)	0.17 (0.15–0.23)	0.27 (0.2–0.36)	0.07
Cholesterol (mg/dL)	193.5 (179- 201.2)	172 (151.2–178) ^*^	176 (155.5-186.7)	NS
Triglycerides (mg/dL)	84 (61-104.2)	86 (67.7-124.2)	97.5 (81.7–117)	NS
HDL (mg/dL)	53 (49.2–66.2)	61.5 (45.5–78)	62 (40.7–72)	NS
LDL (mg/dL)	117.5 (96.5–127)	79.5 (73-103.2)	92.5 (83.7–98)	0.02
Lipid-lowering drugs (number of patients)	0/12	6/12*	6/12*	< 0.001
Number of antihypertensive drugs (median)	0	2.5 (1.7–3.2) ^*^	2 (1–3) ^*^	< 0.001

BMI: Basal Metabolic Index; SBP: Systolic Blood Pressure; DBP: Diastolic Blood Pressure; eGFR: estimated Glomerular Filtration Rate using Chronic Kidney Disease Epidemiology Collaboration (CKD-EPI); HDL: High Density Lipid; LDL: Low Density Lipid, NS: non-significant

Data were expressed as median (range) as they did not show Gaussian distribution and comparisons within and among the groups performed using non-parametric tests (Wilcoxon and Kruskal Wallis, respectively). Statistical significance for all tests was accepted for *p* ≤ 0.05

*- *p*-value < 0.05 vs. Healthy Control

_#_- *p*-value < 0.05 vs. Hypertension

### MSC characterization

MSCs harvested from adipose tissue characterized in normoxia and hypoxia showed plastic-adherent, fibroblast-like morphology (Supplemental Fig S1-A, B) and expressed positive markers CD90, CD73, and CD105 but no significant expression of CD45, CD14, or CD34 markers in either hypoxia or normoxia (Supplemental Figs S2-A, B, S3-A, B). MSCs stained with fluorescently conjugated antibodies against CD73, CD90, and CD105 were strongly positive (Supplemental Fig S4-A). MSCs from all groups showed similar cellular morphology, and their characteristic multilineage potential was confirmed by differentiation into adipocytes, chondrocytes, and osteocytes. There was no difference in trilineage differentiation among the groups. (Supplemental Fig S5-A-C).

### The function of MSCs under normoxic and hypoxic conditions

**Fig. 1:**
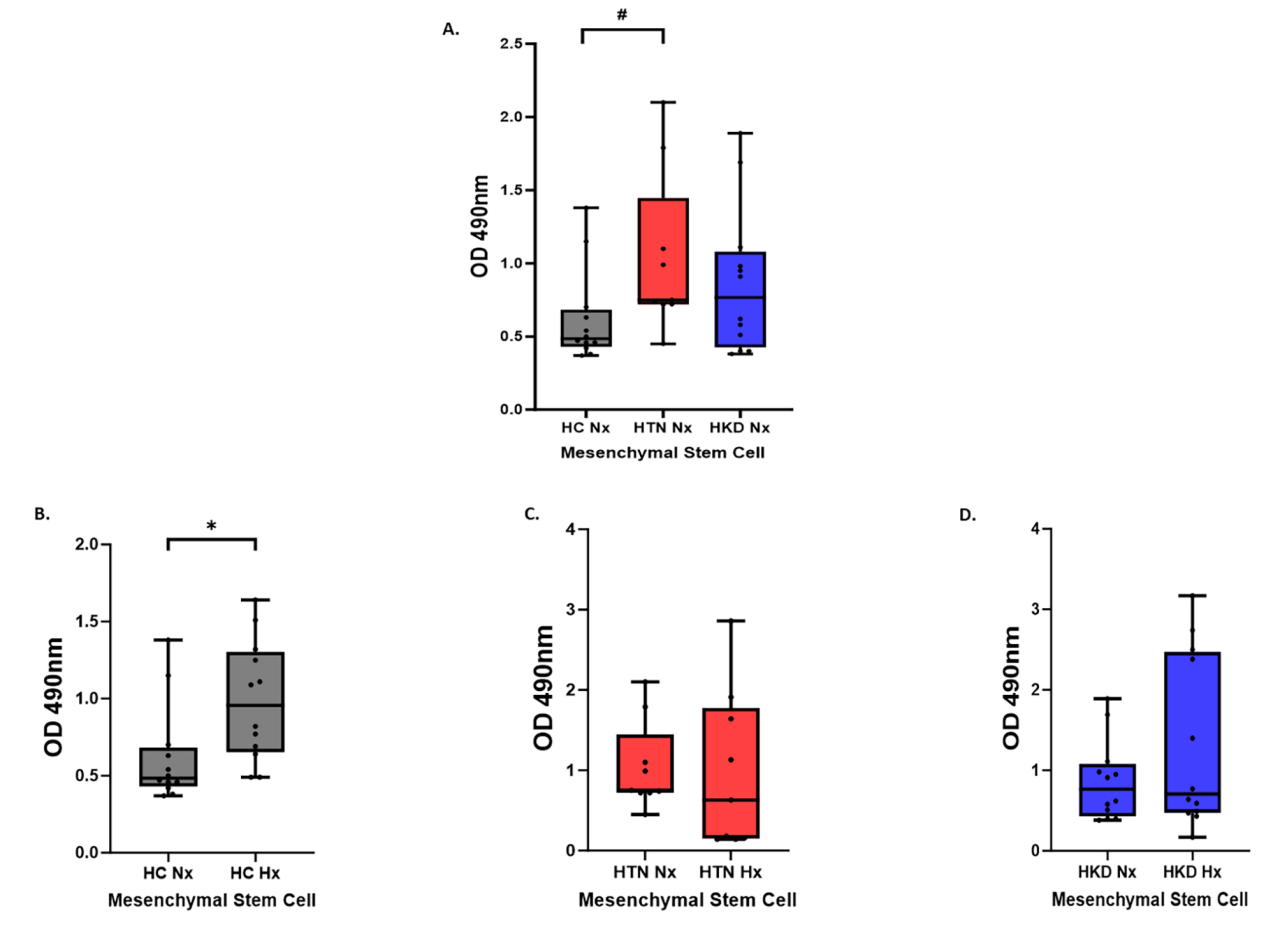
Proliferation. (**A**) Functional differences between Healthy Control (*n* = 12)-, HTN(*n* = 9)-, and HKD(*n* = 12)- MSCs under normoxic and hypoxic conditions. (**B**) Proliferation assay showing effect of HPC on HC-, HTN-, HKD- MSCs A *p* value < 0.05 is considered significant. Significant results represented by: *^#^ *- *p* < 0.05 Hypoxia vs. Normoxia #- *p* < 0.05 Hypertension (HTN) Normoxia vs. Healthy Control Normoxia

At baseline, the proliferative capacity of MSCs was increased in HTN patients in comparison with HC (*p*-value = 0.02). Under hypoxic conditions, the proliferative capacity of HC MSCs increased from baseline (*p*-value = 0.01) (Fig. 1-A) but remained unchanged in the two other groups. As a patient in the HKD were older than the HC and HTN groups we performed a correlation analysis between age and MSC function in normoxic and hypoxic conditions and did not find any significant associations See Supplementary Table 17 A and B.

### MSC secretome studies

#### Inflammatory markers

At baseline normoxic conditions, there was no difference in inflammatory cytokines released among the groups. However, under HPC, there was an increase of IFN- γ levels only in HC-MSCs (*p*-value = 0.04) (Supplemental Figure S12).

#### Angiogenic markers

We investigate the levels of paracrine angiogenic factors secreted by MSCs by measuring VEGF, EGF, and HGF expression in MSCs cultured under normoxic and hypoxic conditions.

**Fig. 2:**
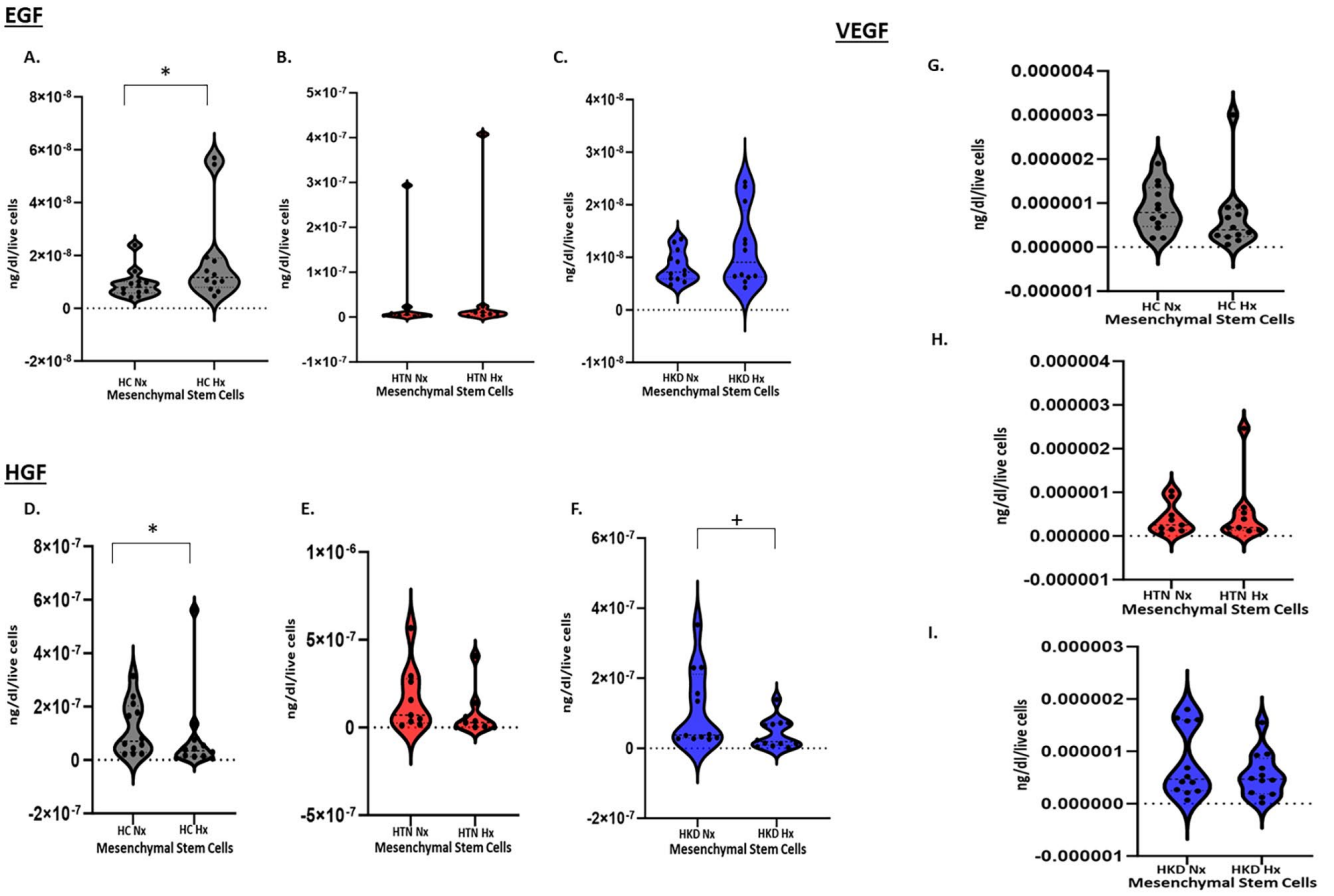
MSC - Secretome Function: ELISA showing the release of pro-angiogenic protein (2A) EGF, (2B) HGF, (2C) VEGF secretome function in Hypoxic vs. Normoxic HC(*n* = 12)-, HTN(*n* = 9)- MSCs, and HKD(*n* = 12) MSCs * *p* < 0.05 Hypoxia vs. Normoxia +- *p* < 0.05 Hypertensive Kidney Disease (HKD) Normoxia vs. Healthy Control

**Fig. 3:**
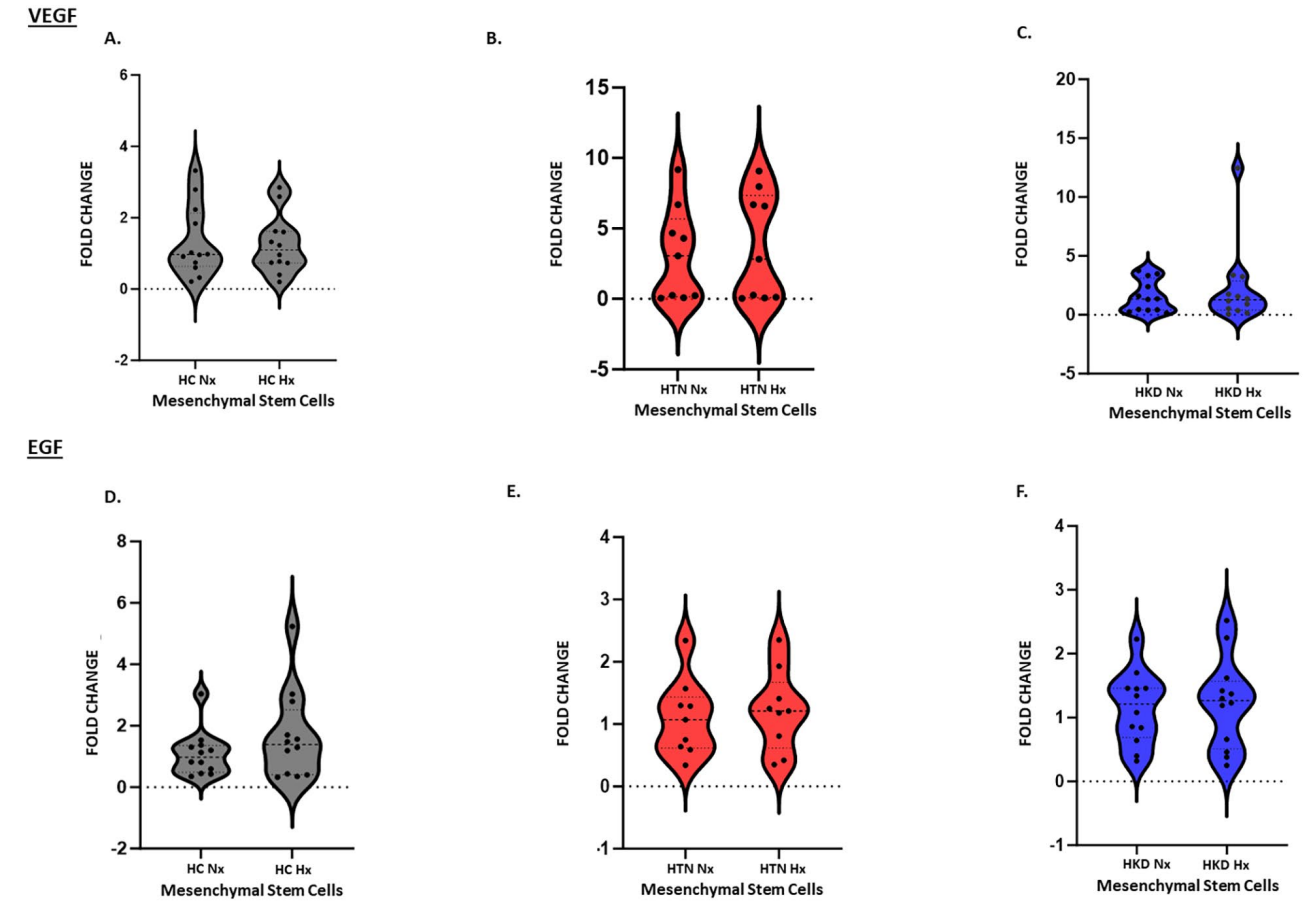
Gene expression of (3A) VEGF & EGF (3B) in Hypoxic vs. Normoxic HC(*n* = 12)-, HTN(*n* = 9)- MSCs, and HKD(*n* = 12) MSCs

Under normoxic conditions at the gene level mRNA level expression of the pro-angiogenic factors VEGF (Fig. 2A) and EGF (Fig. 2B) were similar among the groups and HPC did not exert any significant effect in these specific angiogenic markers at the gene level. Angiogenesis secretome studies at baseline normoxic conditions did not show with any differences between the groups. However, under HPC, while there was no significant change in VEGF secretion there was an increase in secretion of EGF in HC-MSCs (*p*-value = 0.04) (Fig. 3A-A). Furthermore, there was a decrease in the secretion of HGF in HC-MSCs (*p*-value = 0.04) (Fig. 3B-A) as well as in HKD-MSCs (*p*-value = 0.04) (Fig. 3B-C) as compared to baseline.

#### PCR markers

Under normoxic conditions at the gene level expression TGF-β, PGE, iNOS, GAPDH, IDO, IL10, IL4 were similar among the groups during normoxia and HPC did not exert any significant effect (Supplementary Table S3).

### Effects on senescence

**Fig. 4:**
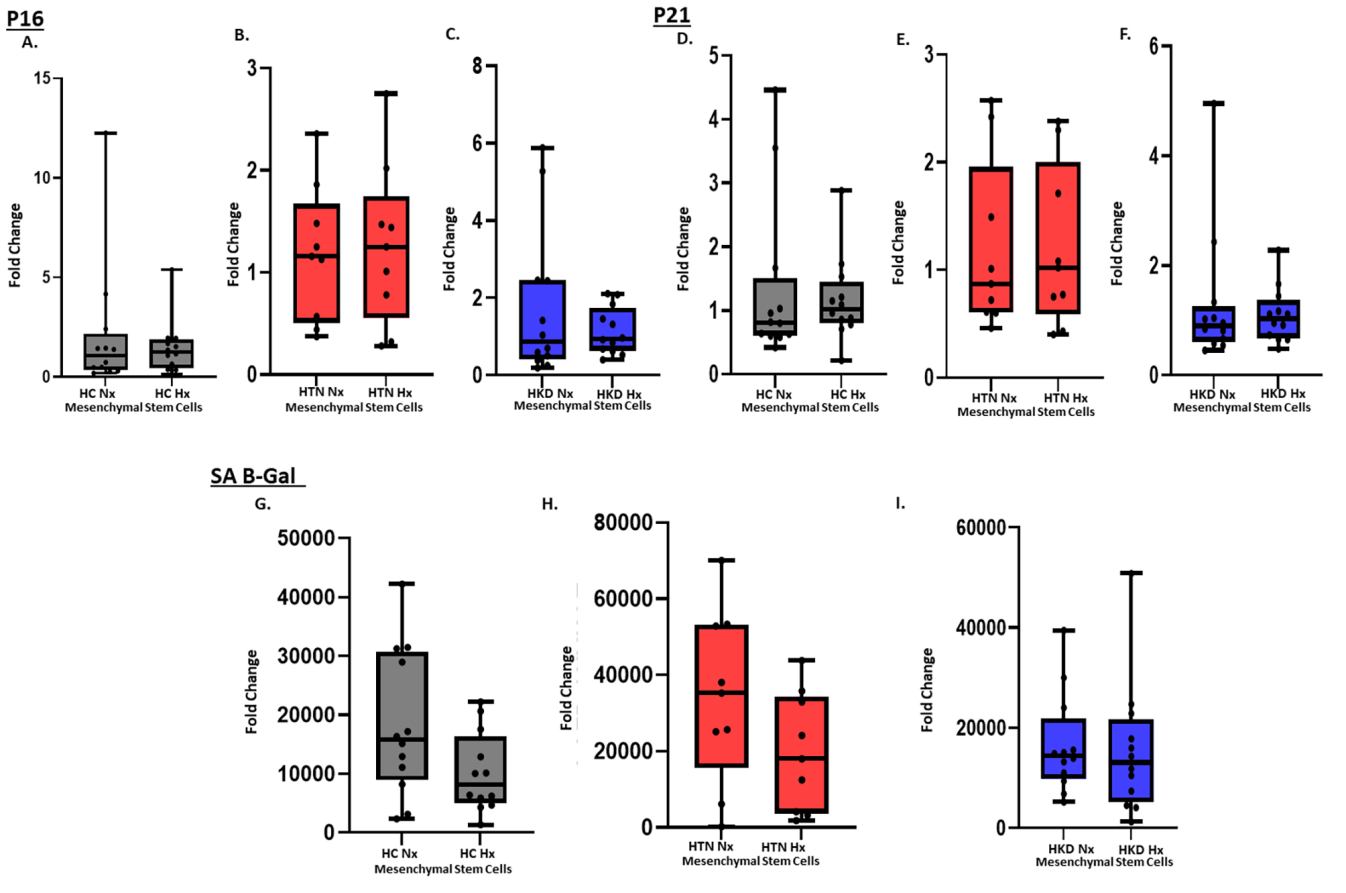
mRNA expression: Fold change, GAPDH. Gene expression of (4 A) p16 & (4B) p21 in HC(*n* = 12)-, HTN(*n* = 9)-, and HKD(*n* = 12)- MSCs in hypoxia vs. normoxia conditions. SA-β-gal assay showing senescence(4 C) in Hypoxic vs. Normoxic HC(*n* = 12)-, HTN(*n* = 9)- MSCs, and HKD(*n* = 12) MSCs * *p* < 0.05 Hypoxia vs. Normoxia

Neither under normoxic or hypoxic conditions, senescence gene level expression of p16 (Fig. 4A) and p21 (Fig. 4B) were not significantly different in MSCs between the groups. While senescence-associated β-galactosidase (SA-β-gal) activity was similar between the groups at baseline and under HPC. HPC tended to decrease the SA-β-gal activity in HC-MSCs (*p*-value = 0.06) (Fig. 4C-A).

### Transcriptomic analysis

#### Normoxic hypertensive kidney disease MSCs vs. healthy control MSCs

**Fig. 5:**
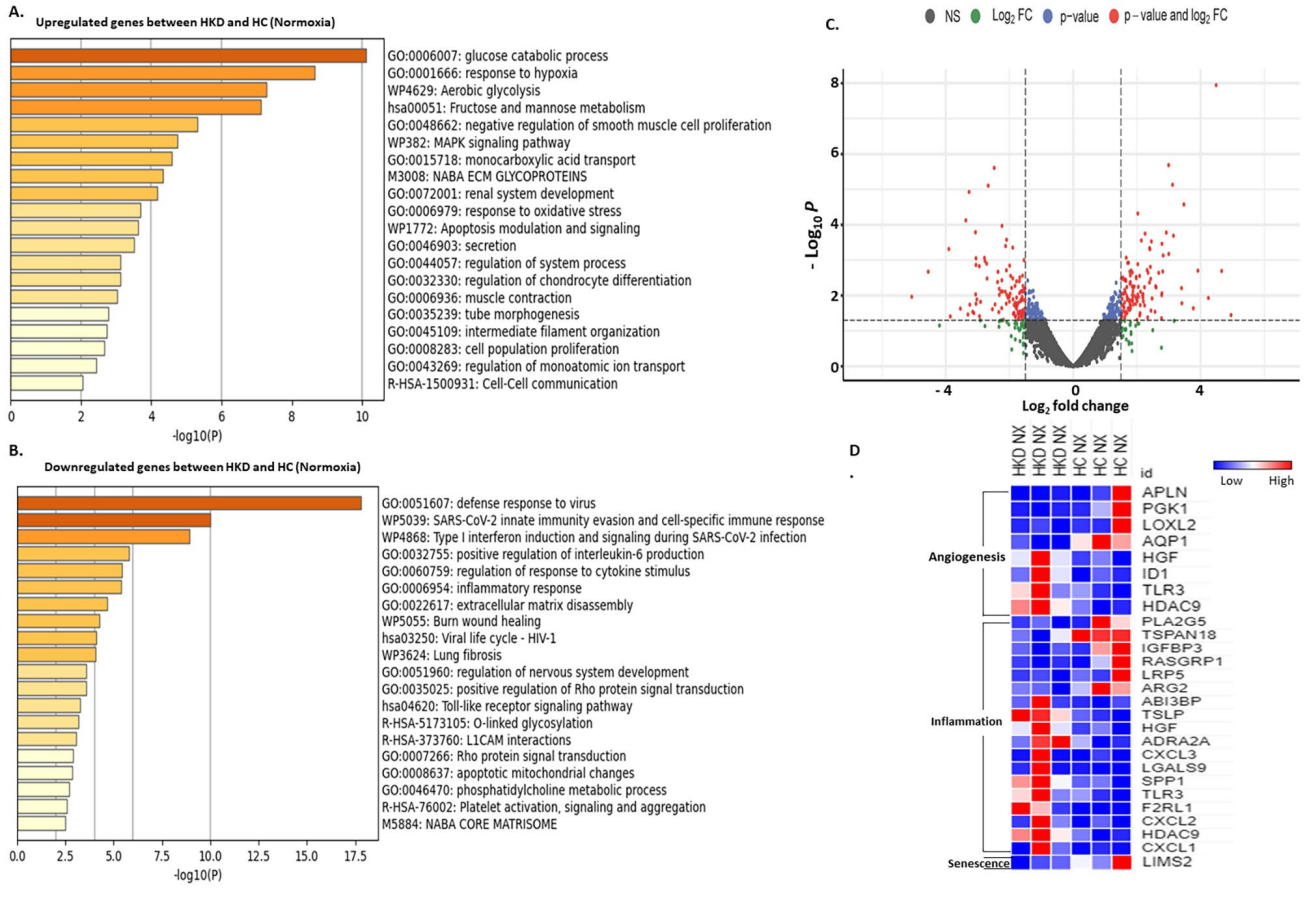
Normoxia HKD vs. normoxia HC MSCs. Gene ontology (GO) analysis of hallmark and biological process of upregulated (**A**) and downregulated (**B**) gene sets with significant changes between normoxia HKD and normoxia HC MSCs. Volcano plot of dysregulated genes between normoxia HKD and normoxia HC MSCs (**C**). The vertical axis (y-axis) corresponds to the -log (*p*-value) and the horizontal axis (x-axis) displays the log-fold change value. Genes with higher and lower levels between normoxia HKD and normoxia HC MSCs are shown with red and blue dots, respectively, while non-significant genes are shown as grey dots. Specifically, red dots represent significance with the *p*-value and log2 FC, blue dots represent significance with *p*-values only, and the green dots represent significance with the log2 FC values. Heat map of genes with significant changes in normoxia HKD vs. normoxia HC MSCs filtered by function for angiogenesis, inflammation, and senescence (**D**)

Normoxic-MSCs of hypertensive kidney disease vs. healthy control mapped a total of 13,493 genes, with 401 significant dysregulated genes (*n* = 237 upregulated & *n* = 164 downregulated). Gene ontology analysis showed that genes upregulated were implicated in the modulation of inflammation, response to hypoxia, proliferation, and apoptosis, and those downregulated participated in mitochondrial apoptosis, inflammatory response, positive regulation of interleukin-6 production, and platelet activation (Fig. 5A, B **respectively)**. The volcano plot demonstrated the distribution of differentially expressed genes, with downregulated and upregulated genes based on *p*-value and log_2_fc (Fig. 5-C). Heatmap showed somewhat equal upregulation of significantly dysregulated angiogenetic genes in HKD and HC MSCs. Inflammatory genes were increased in HKD MSCs than in HC MSCs whereas, LIMS2, a senescent gene was more prominently expressed in HC MSCs as compared to HKD MSCs. (Fig. 5-D).

#### Normoxic hypertensive kidney disease MSCs vs. normoxic hypertensive MSCs

**Fig. 6:**
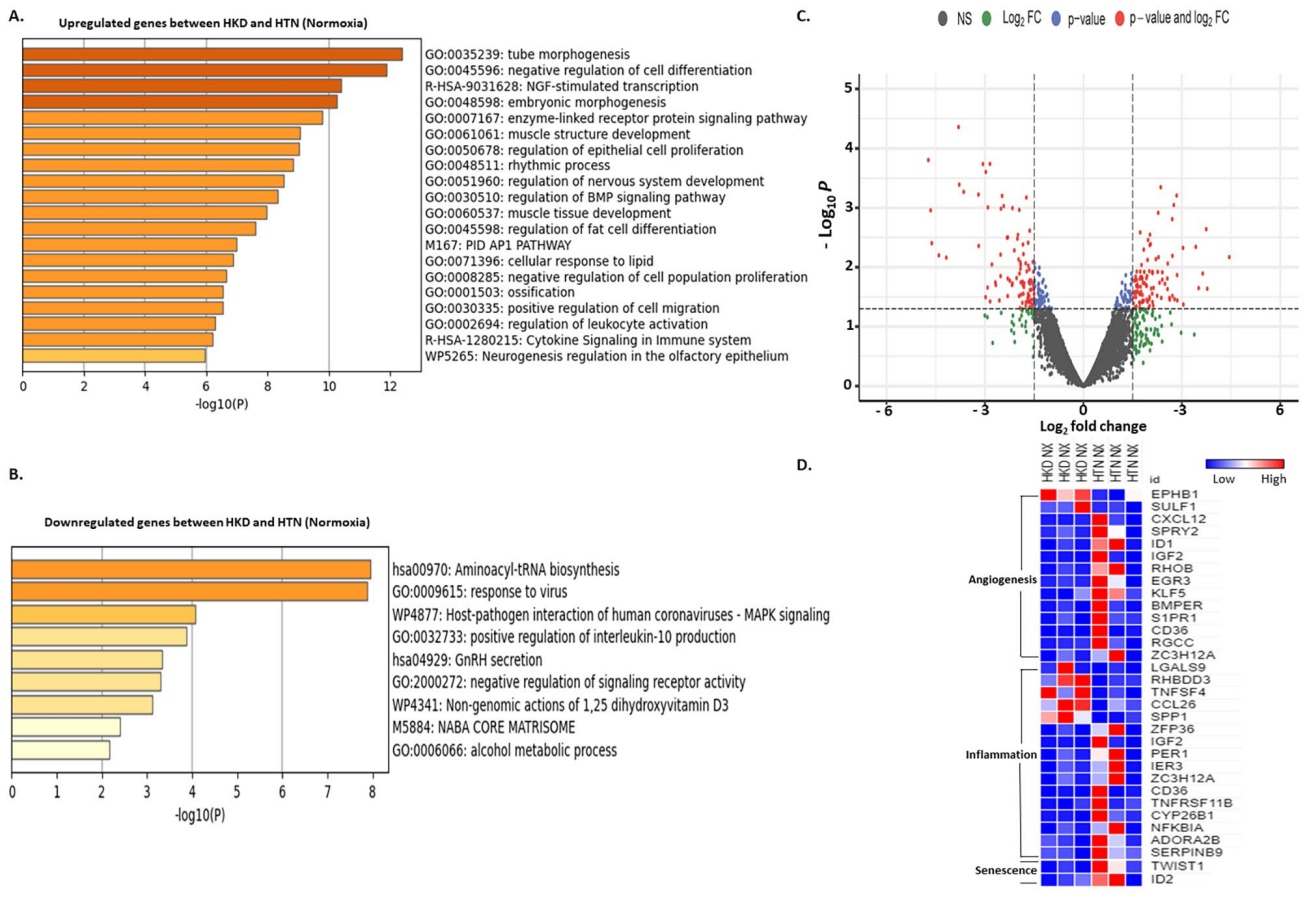
Normoxia HKD vs. normoxia HTN MSCs. Gene ontology (GO) analysis of hallmark and biological process of upregulated (**A**) and downregulated (**B**) gene sets respectively with significant changes between normoxia HKD and normoxia HTN MSCs. Volcano plot of dysregulated genes between normoxia HKD and normoxia HTN MSCs (**C**). The vertical axis (y-axis) corresponds to the -log (*p*-value) and the horizontal axis (x-axis) displays the log-fold change value. Genes with higher and lower levels between normoxia HKD and normoxia HTN MSCs are shown with red and blue dots, respectively, while non-significant genes are shown as grey dots. Specifically, red dots represent significance with the *p*-value and log2 FC, blue dots represent significance with *p*-values only, and the green dots represent significance with the log2 FC values. Heat map of genes with significant changes in normoxia HKD vs. normoxia HTN MSCs for angiogenesis, inflammation, and senescence (**D**)

Normoxic-MSCs of hypertensive kidney disease vs. hypertensive MSCs mapped a total of 13,675 genes, with 282 significant dysregulated genes (*n* = 191 upregulated & *n* = 91 downregulated). Gene ontology analysis showed that genes upregulated were involved in the modulation of proliferation, migration, and embryonic morphogenesis, but those downregulated participated in inflammation and positive regulation of interleukin 10 production (Fig. 6-A, B **respectively)**. The volcano plot demonstrated the distribution of differentially expressed genes, with downregulated and upregulated genes based on *p*-value and log_2_fc (Fig. 6C). Heatmap showed upregulation of significant differential expressed angiogenetic, inflammatory, and senescence genes more in HTN MSCs than in HKD MSCs (Fig. 6D).

#### Normoxic- vs. hypoxic- MSCs hypertensive kidney disease

**Fig. 7:**
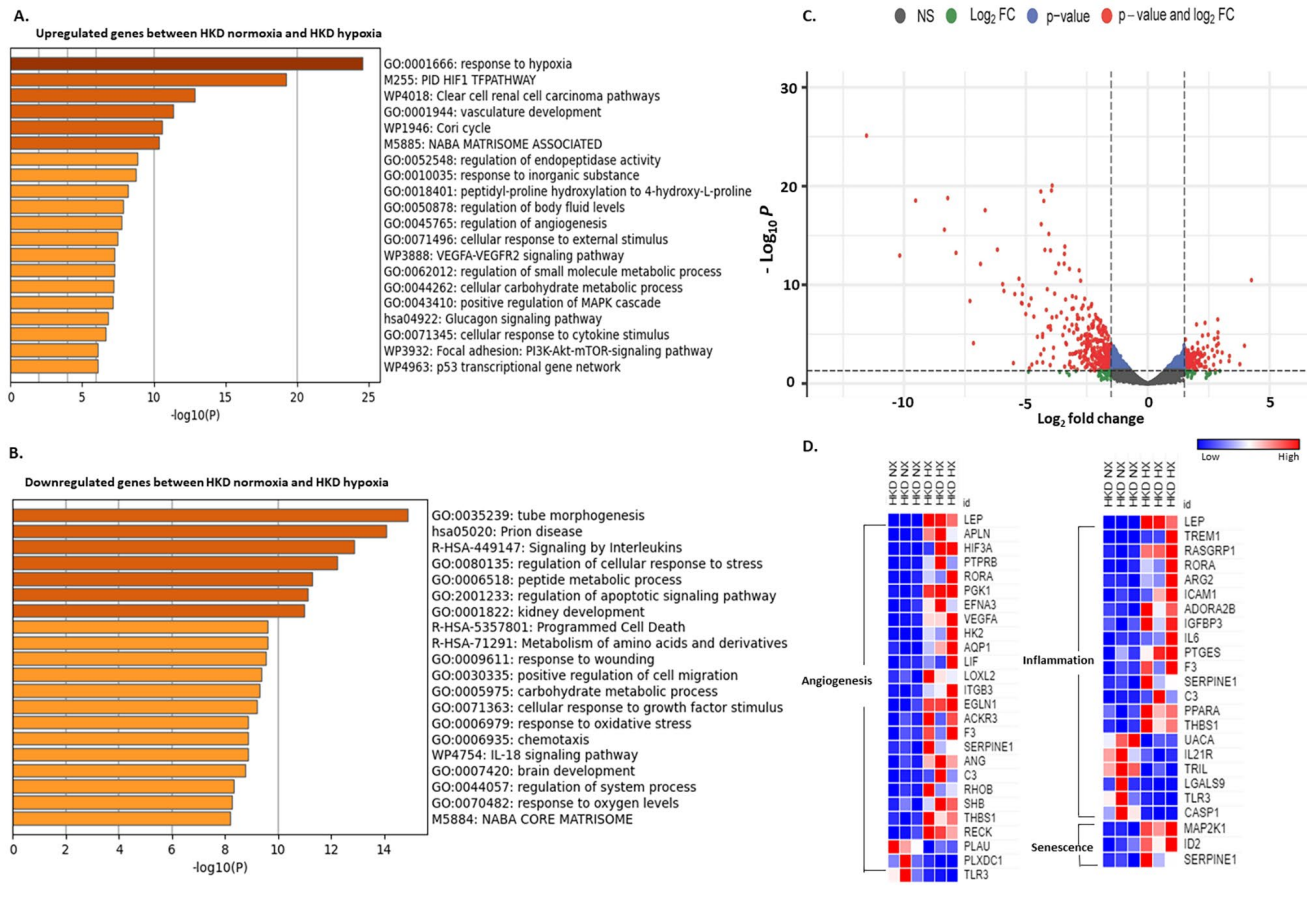
Normoxic HKD vs. hypoxic HKD MSCs. Gene ontology (GO) analysis of hallmark and biological process of upregulated (**A**) and downregulated (**B**) gene sets respectively with significant changes between normoxic HKD and hypoxic HKD MSCs. Volcano plot of dysregulated genes between normoxic HKD and hypoxic HKD MSCs (**C**). The vertical axis (y-axis) corresponds to the -log (*p*-value) and the horizontal axis (x-axis) displays the log-fold change value. Genes with higher and lower levels between normoxic HKD and hypoxic HKD MSCs are shown with red and blue dots, respectively, while non-significant genes are shown as grey dots. Specifically, red dots represent significance with the *p*-value and log2 FC, blue dots represent significance with *p*-values only, and the green dots represent significance with the log2 FC values. Heat map of genes with significant changes in normoxia HKD vs. hypoxic HKD MSCs for angiogenesis, inflammation, and senescence (**D**) HC: Healthy control; HTN: Hypertension; HKD: Hypertensive Kidney disease; MSC: Mesenchymal Stem Cells; IFN- γ: Interferon gamma; IL-1α: Interleukin-1 alpha; IL-6: Interleukin 6; IL-8: Interleukin 8; TNF-α: Tumour necrosis factor α; VEGF: Vascular endothelial growth factor; EGF: Epidermal growth factor; HGF: Hepatocyte Growth Factor; SA-β-gal: Senescence-associated beta-galactosidase

Similarly, in HKD-MSCs, a total of 13,581 genes were mapped, with 1375 significant dysregulated genes (*n* = 496 upregulated & *n* = 879 downregulated). Gene ontology analysis showed genes upregulated were involved in the modulation of pro-angiogenic functions and vasculature development, but those downregulated participated in the apoptotic pathway, positive regulation of cell migration, and response to oxygen levels (Fig. 7A, B). The volcano plot demonstrated the distribution of differentially expressed genes, with downregulated and upregulated genes based on *p*-value and log_2_fc (Fig. 7C). Heatmap showed dysregulated genes significant for angiogenesis, inflammation, and senescence upregulated more in hypoxic HKD MSCs compared to normoxic HKD MSCs (Fig. 7D).

### Gene and transcription factor interaction networks

#### Normoxic hypertensive kidney disease MSCs vs. healthy control MSCs

STRING analysis revealed several interactions among the genes and transcription factors between HKD and HC MSCs in normoxic conditions suggesting genes are fairly regulated by transcription factors (Supplemental Fig. 9).

#### Normoxic hypertensive kidney disease MSCs vs. normoxic hypertensive MSCs

STRING analysis revealed numerous interactions among the genes and transcription factors between HKD and HTN MSCs in normoxic conditions indicating that gene regulation is significantly influenced by transcription factors (Supplemental Fig. 10).

#### Normoxic- vs. hypoxic- MSCs hypertensive kidney disease

The STRING analysis revealed extensive interactions between genes and transcription factors between HKD MSCs in normoxia and hypoxia suggesting gene expression is highly regulated by transcription factors (Supplemental Fig. 11).

## Discussion

The current study shows that HPC alters human adipose tissue derived MSC functional properties including proliferative capacity with an increase of specific pro-angiogenic genes and proteins as well as inflammatory genes. HPC effects seemed to be more favorable in MSCs from healthy individuals than in patients with hypertension with or without kidney disease, as reflected by increased proliferation and EGF secretion. Hypoxia does not seem to affect senescence significantly in any group, but there was a trend in decreased senescence in the HC group. Transcriptome analysis of MSCs at baseline normoxic conditions showed that gene sets modulating vasculature development, proliferation, and response to growth factors were mostly upregulated among the groups. Gene sets upregulating pro-angiogenic function was enhanced after HPC in HC and HKD groups, however, did not have a significant effect in hypertensive patients.

We previously have shown that MSCs from patients with ARAS and with CKD presented impaired functionality as compared to HC MSCs, as evidenced by decreased expression of angiogenic proteins, reduced migration capacity, and DNA damage [31]. Similar findings were also observed in MSCs in pre-clinical ARAS porcine model [12]. In our current study, MSCs obtained from the patients with hypertension without kidney disease expressed increased proliferation compared to healthy controls but this was not different from HKD patients. This might be related to the fact that our cohort of patients with HKD, despite being older hypertensive patients with dyslipidemia had only mild to moderate CKD with no other major comorbidities. In addition, most of the patients with HTN and HKD were under treatment with blood pressure medications and lowering lipid-lowering agents (e.g., statins) which potentially could have affected the MSC functionality. A study by Ronit Lavi and colleagues has shown that statin supplementation improved the renal microenvironment and decreased apoptosis in endothelial progenitor cells (EPC) suggesting the pleiotropic effects of statins [32–34]. Although the baseline proliferative capacity of HC MSCs was not different from patients with HKD, once cultured under hypoxia, the proliferation rate only increased in HC MSCs. This might be related to disease processes causing abnormal MSCs and potentially reducing their proliferative capacity which might explain the lack of response to HPC of MSCs from patients with HTN and HKD [35, 36].

MSCs also may affect the microenvironment upon injury, promoting cytoprotection, angiogenesis, and tissue repair of the damaged area throughout paracrine properties [37]. We looked at specific inflammatory markers and there was no significant effect on inflammation at the secretome level except increased IFN-γ secretion in HC MSCs during HPC when compared to normoxic conditions. This is an interesting finding as a recent study demonstrated that MSCs and their secretome under IFN-γ and hypoxic stimulation induced the highest angiogenic properties of human umbilical vein endothelial cells (HUVECs) enhancing their efficacy by increasing tube formation and wound healing. This revealed the higher angiogenic potential of HUVECs in the presence of IFN-γ conditioned MSC secretome [38]. Hypoxic preconditioning can also induce the expression of stress proteins, such as heat shock proteins, which can trigger an immune response and inflammation [39]. While hypoxic preconditioning has been shown to have a variety of beneficial effects, its effects on inflammation are still a subject of research [40]. Furthermore, we look at MSCs’ angiogenic properties. We and others have shown that HPC exerts beneficial effects on angiogenic protein expression [31, 41]. In our previous pre-clinical study in a porcine model of ARAS, specific pro-angiogenic genes assessed by RT-PCR were lower at baseline but increased under HPC and once MSCs were cultured under hypoxia. The expression of VEGF and EGF was significantly increased as compared to normoxia [12]. This same effect was seen in human MSCs from patients with ARAS. HPC improved VEGF secretion in MSCs from patients with ARAS but decreased HGF secretion [31]. In our current study, while there was no increase of specific pro-angiogenic genes in any of the groups, specifically VEGF and EGF, assessed by RT-PCR when assessed at the mRNA level by RNA sequencing, we observed upregulation of genes VEGF in HKD and HGF in HTN and HKD but there was no difference at the secretome level. However, we did observe enhanced EGF secretion in MSCs from healthy control patients stimulated by HPC by RT-PCR. Assessing EGF levels in bodily fluids, like blood, saliva, and urine can offer insights into the degree of tissue damage and the ongoing healing process. Steven Menez and colleagues showed elevated levels of urinary EGF to creatinine ratio (uEGF/Cr) during the postdischarge period following an acute kidney injury (AKI) event, suggesting a sign of adaptive repair [42]. Yet, HGF decreased in the conditioned media of HC and HKD MSCs once exposed to HPC, with no effect on HTN MSCs. The reason for the lack of enhanced VEGF and HGF secretion is not clear. Hypoxia induces different responses in MSCs by hypoxia-inducible factor-1 (HIF-1) activation [43]. The cellular response to hypoxia is conferred to a large extent by activation of the hypoxia-sensitive transcription factor. The target genes of HIF-1 increase oxygen transport, for instance, through VEGF-induced angiogenesis, improving tissue function at low oxygen availability [44]. HGF is an important cytokine for angiogenesis, anti-inflammation, and anti-apoptotic stimulates endothelial cell motility and growth, which also enhances VEGF-induced angiogenesis [45, 46]. HGF is mostly enhanced during HPC, but some studies have shown decreased expression of HGF under HPC, especially in aged cells as demonstrated in MSCs from older patients with ARAS [31, 47, 48].

VEGF plays an important role in angiogenesis. HPC increases VEGF concentration in conditioned media [12, 47], which is assumed to be related to the regenerative capacities of MSCs [49]. Other factors like HGF and EGF also influence the therapeutic effect of MSCs [50]. Hypoxic preconditioning may alter the expression of certain genes and signaling pathways involved in angiogenesis, leading to a decreased response to pro-angiogenic stimuli especially in aged cells. Our results can be advocated by the studies by Hayashi and colleagues [51] which demonstrated that vascular cells when treated with hypoxia downregulate HGF production due to decreased cAMP, consistent with their potential role in the pathophysiology of ischemic diseases. Another element worth noting is the protocol used for HPC. Some studies proposed that HPC for 24 h rather than 48 h appears to be optimal for umbilical cord-derived MSCs [52]. Overstretched cells are perhaps not optimal for exerting desirable paracrine effects. The survival rate of MSCs decreases even further at 72 h with 1% O_2_ as compared to 24 and 48 h suggesting that excessive HIF-1α expression negatively affects cell survival [53].

Another important assessment for regenerative capacity is the degree of senescence of the MSCs. Senescent cells can release factors that cause inflammation and can cause dysfunctional MSCs, accumulating with aging at etiological sites in multiple chronic diseases [54]. At the molecular level, upregulation of the cyclin-dependent kinase inhibitors p16 and p21 mediates senescence [55]. We found that senescence genes p16, p21, and senescence-associated β-galactosidase activity had little to no effect under HPC throughout the groups. Only HC MSCs tended to decrease SA-β-gal activity (*p*-value = 0.06). In our study, senescence markers were not different between groups at baseline. It is possible that, despite patients with HKD being older, the lack of major comorbidities and use of potential pleiotropic drugs might have contributed to the lack of differences among the groups, but overall young and healthy patients seem to benefit more from HPC effects on mitigating MSC senescence.

At the gene level, in HTN MSCs, genes HGF and EGR3 involved with angiogenesis were modestly upregulated in normoxia along with upregulation of genes CXCL1, CXCL2, CXCL3, CXCL5 compared to HC which act as a chemoattractant for immune cells and play an important role in the regulation of immune and inflammatory response [56]. This potentially could contribute to inflammation leading to hypertension [57]. Furthermore, there is upregulation of senescent regulator gene ID2 in HTN MSCs which negatively regulates transcription factors by forming heterodimers and inhibiting their DNA binding and transcriptional activity regulating cell differentiation [58]. In normoxia at the gene levels, HKD versus HC MSCs were found to have almost equal angiogenic genes but more inflammatory-associated genes in HKD than HC MSCs as ARG2 and IGFB3 are also related to angiogenesis. Hypoxia upregulated the angiogenic genes in HC MSCs and downregulated the inflammatory genes involved in the cellular response to TNF. In HKD MSCs, hypoxia upregulated angiogenic and inflammatory genes as compared to normoxia. Specific genes like ZFP36 and RORA are negative regulators of inflammation associated with anti-inflammatory environment and they were upregulated in HC and HKD MSCs during hypoxia, respectively. Interestingly, in HKD MSCs, HPC increased pro-angiogenetic genes such as VEGF-A at the gene level, but this did not translate to an increase at the secretome level. This could be stipulated due to post-transcriptional modifications or protein processing [59]. As the regulatory mechanism is specific to each gene, a gene may be transcribed but not translated, or it may be translated but the resulting protein may not be secreted. These regulatory mechanisms can result in differences between the transcriptome and secretome. It needs to be taken into consideration that gene expression changes upon aging. Additionally, string analysis revealed numerous interactions among differentially expressed genes and transcription factors between HKD normoxia and hypoxia, suggesting that transcription factors potentially are a big player regulating gene expression. Conversely, fewer interactions were observed between HKD and HC under normoxic conditions, as well as between HKD and HTN under normoxic conditions, indicating a potentially reduced role of transcription factors in regulating gene expression. The differences between the functions of MSCs and the results of RNA sequencing after hypoxic preconditioning can be attributed to several factors. These include biological variation among MSCs, experimental variability, time-dependent effects of hypoxia, variations in analytical methods, and potential limitations of RNA sequencing in capturing all cellular adaptations.

Hence, the molecular sequel of aging varies between different regulatory networks, cell types, environments, and cellular fates. However, it’s worth noting that the effects of hypoxic preconditioning on angiogenesis may depend on the specific tissue or organ being studied, as well as the severity and duration of the hypoxic stimulus. The duration of disease and aging of MSC may not affect the overall functionality of MSC and needs further understanding [60]. Overall, more research is needed to fully understand the mechanisms underlying this phenomenon. HTN can impair MSC function and differentiation, reduce their immunomodulatory abilities, contribute to endothelial dysfunction, promote premature senescence of MSCs, and alter the microenvironment in which MSCs reside. These effects can hinder the regenerative capacity of MSCs and their ability to repair tissues. The specific impact on MSCs may vary depending on factors such as the severity and duration of hypertension and individual health status. MSCs derived from patients with HKD exhibit differences compared to MSCs from HTN due to factors such as the unique microenvironment within the kidneys of HKD patients including renal tissue damage with fibrosis, altered signaling pathways, epigenetic modifications, and the presence of systemic factors and comorbidities. These factors contribute to variations in the behavior, functional properties, and gene expression patterns of MSCs in HKD.

This study showed for the first time that the baseline characteristics of the MSCs from patients with HKD, with no other major comorbidity, do not differ as much from HC and hypertensive patients. In addition, HPC did not improve the effectiveness of MSC functionality drastically as compared to normoxia, specifically among the HKD group. This study is limited by the number of participants. Therefore, further basic, and translational studies are needed to continue exploring the potential therapeutic applications of hypoxic preconditioning to improve the functioning of MSC.

In conclusion, hypoxic preconditioning seems to affect human MSC functionality modestly but warrants further human-based studies for better understanding. At the gene level, HPC leads to the upregulation of angiogenic genes but there was also an increase in a few inflammatory and senescence genes which require further exploration. Integrating RNA sequencing data with other functional and proteomic analyses is crucial to gain a comprehensive understanding of MSC behavior and functional outcomes. Moreover, our findings have important implications for the self-healing capacity of healthy control MSCs rather than the MSCs of HTN and HKD groups. Therefore, HPC-enhanced healthy MSCs can be potentially used for allogenic transfers considering their low immunogenicity. Future studies are needed to explore MSC characteristics in different disease settings and to identify measures such as pre-conditioning to preserve MSC function.

### Electronic supplementary material

Below is the link to the electronic supplementary material.

Supplementary Material 1

## Data Availability

All the data generated or analyzed in our study, including raw data of histology findings are available upon request.

## Abbreviations

Nx: Normoxia
Hx: Hypoxia
HC: Healthy Control
HTN: Hypertension
HKD: Hypertensive kidney disease
HPC: Hypoxia preconditioning
MSC: Mesenchymal Stem/Stromal cells
BMI: Basal Metabolic Index
SBP: Systolic Blood Pressure
DBP: Diastolic Blood Pressure
HDL: High Density Lipid
LDL: Low Density Lipid
eGFR: Glomerular filtration rate
EGF: Epidermal Growth Factor
VEGF: Vascular Endothelial Growth Factor
HGF: Hepatocyte Growth FactorIL-1α:IL Interleukin 1-alpha
IFN-γ: Interferon-gamma
TNF-α: Tumor Necrosis Factor-alpha
IL-6: Interleukin 6
MCP-1: Monocyte Chemoattractant Protein-1
IL-8: Interleukin-8
