# Supplementary material for: The impact of hypoxia preconditioning on mesenchymal stem cells performance in hypertensive kidney disease
Source: Stem Cell Res Ther. 2024 Jun 9;15:162. doi: 10.1186/s13287-024-03778-1 (PMC11163800; doi:10.1186/s13287-024-03778-1)
Supplement: Supplementary file 1 — Supplementary Material 1 [file 13287_2024_3778_MOESM1_ESM.pdf]

SUPPLEMENTAL TABLE OF CONTENTS

Supplemental Method:

Gating strategy for flow Cytometry to confirm the expression of MSC-specific surface markers.

Using the aspect ratio intensity and area of the brightfield channel on the Amnis Flow sight we were able to visualize and gate the single cells. Thresholds on the Amnis flow sight were determined by histograms generated by single color stained positive and negative beads. Using this data, baseline thresholds were determined to run our samples. The threshold generated by the control beads was used to visualize the images of cells in a scatterplot and determine the single cells that were positively stained[1].

Supplemental Figures

Supplemental Figure 1: Mesenchymal stem cell (MSC) showing typical morphological appearance in normoxic and hypoxic conditions under the microscope as spindle-shaped, fibroblast-like cells in culture. **(A, B respectively)**

Supplemental Figure 2: Mesenchymal stem cell (MSC) characterization in normoxia conditions. **(A)** Positive MSC markers (CD73, CD90 and CD105) and **(B)** negative MSC markers (CD14, CD34, CD45).

Supplemental Figure 3: Mesenchymal stem cell (MSC) characterization in hypoxia conditions. **(A)** Positive MSC markers (CD73, CD90 and CD105) and **(B)** negative MSC markers (CD14, CD34, CD45).

Supplemental Figure 4: MSC characterization by with fluorescently conjugated antibodies against CD73, CD90, CD105. (A) . Cells strongly positive for markers CD73, CD90 and CD105.

Supplemental Figure 5: MSC Morphology and differentiation. Tri-lineage differentiation into adipocyte (FABP4), chondrocyte (Aggrecan) and osteocytes (osteocalcin) and their quantification.

Supplemental Figure 6: Normoxia HTN vs Normoxia Healthy Control MSCs. Normoxic-MSC of hypertension vs. healthy control mapped a total of 13,469 genes, with 463 significant dysregulated genes (n=306 upregulated & n=157 downregulated).

Gene ontology analysis showed that upregulated genes were implicated in modulation of vasculature development, migration, response to growth factor and negative regulation of proliferation, whereas downregulated genes participated in insulin like growth factor binding proteins **(A, B, respectively)**. Volcano plot demonstrated the distribution of differentially expressed genes, with downregulated and upregulated genes based on p-value and log_2fc **(C)**. Heatmap showed angiogenic and inflammatory genes upregulated in HTN MSCs compared to HC MSCs and an equal dysregulation of senescence genes in MSCs of both the groups **(D)**.

Supplemental Figure 7: Normoxia Healthy Control vs Hypoxia Healthy Control MSCs. In healthy control-MSCs, a total of 13138 genes were mapped, with 261 significant dysregulated genes (n=60 upregulated & n=201 downregulated). Gene ontology analysis showed that gene upregulated participated in modulation of angiogenesis and methylation, whereas downregulated genes were involved in cellular response to tumor necrosis factor **(A, B, respectively)**. Volcano plot demonstrated the distribution of differentially expressed genes, with downregulated and upregulated genes based on p-value and log_2fc **(C)**. Heatmap showed dysregulated genes significant for angiogenesis, and inflammation upregulated more in hypoxic HC MSCs compared to normoxic HC MSCs **(D)**.

Supplemental Figure 8: Gene ontology (GO) analysis pathways (A) Biological Process (B) Molecular Functions (C) Cellular Components of Upregulated gene sets with significant changes between Hypoxia HKD and Normoxia HKD MSCs.

Supplemental Figure 9: Genes and Transcription Factor (TF) network Interactions between Normoxia HKD and Normoxia HC mRNA targets derived from STRING. Color lines represent interactions between Normoxia HKD and Normoxia HC mRNA targets according to the functional association networks. Red circles indicate common (TF) mRNA targets between Normoxia HKD MSCs and Normoxia HC MSCs**.**

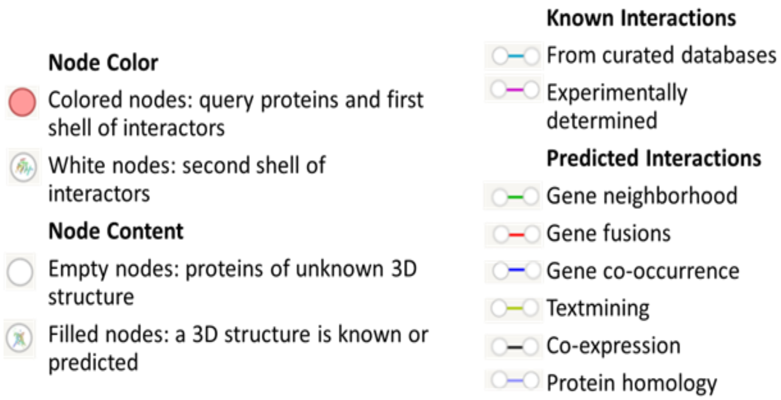

Supplemental Figure 10: Genes and Transcription Factor (TF) network Interactions between Normoxia HKD and Normoxia HTN mRNA targets derived from STRING. Color lines represent interactions between Normoxia HKD and Normoxia HTN mRNA targets according to the functional association networks. Red circles indicate common (TF) mRNA targets between Normoxia HKD MSCs and Normoxia HTN MSCs**.**

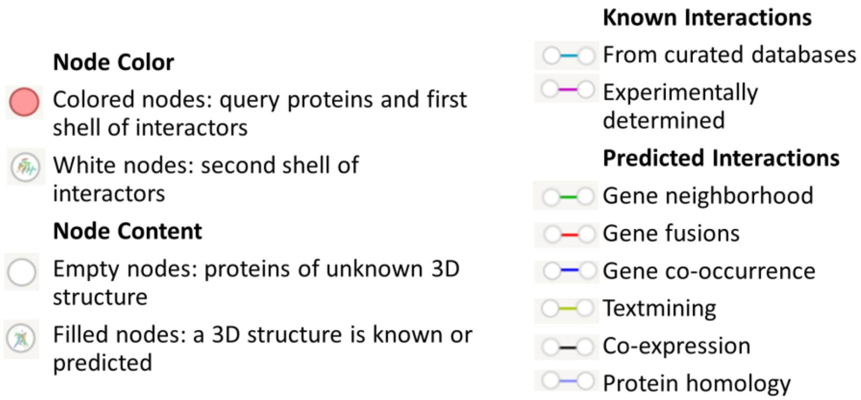

Supplemental Figure 11: Genes and Transcription Factor (TF) network Interactions between HKD normoxia and hypoxia mRNA targets derived from STRING. Color lines represent interactions between HKD normoxia and hypoxia mRNA targets according to the functional association networks. Red circles indicate common (TF) mRNA targets between HKD normoxia and hypoxia**.**

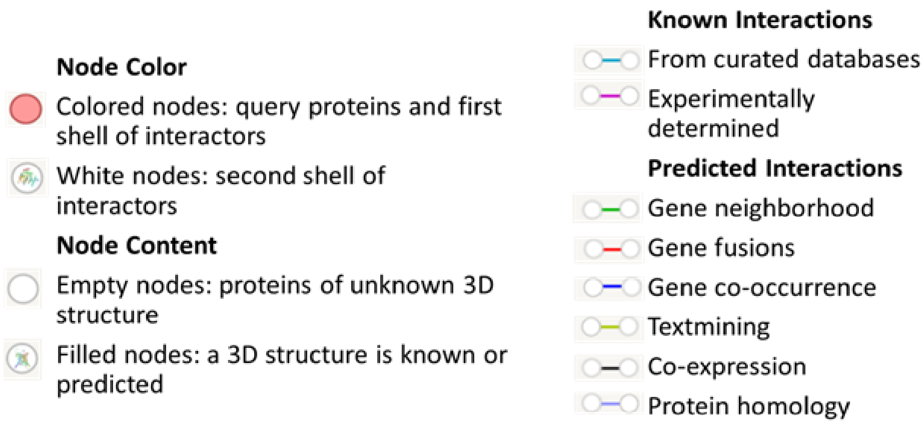

Supplemental Figure S12: Inflammatory markers

Inflammatory factors under normoxic and hypoxic state in IFN- γ, IL-1α, IL-6, IL-8, and TNF-α in HC(n=12)-, HTN(n=9)- MSCs, and HKD(n=12)- MSCs.At baseline normoxic conditions, there was no difference in inflammatory cytokines released among the groups **(Fig S12A-E)**. However, under HPC, there was an increase of IFN-γ levels only in HC-MSCs (p-value=0.04**) (Fig S12-A).** Among the baseline of the HC, HTN and HKD, there was no difference **(Fig S13).**

Supplemental Table S1: Angiogenesis secretome markers

Under normoxic conditions the expression of the pro-angiogenic factors VEGF and EGF were similar among the groups and HPC did not exert any significant effect in these specific angiogenic markers. There was a significant increase in HC MSCs EGF and HGF (Elisa)after hypoxia. We also saw a significant decrease in HKD MSCs HGF after hypoxia.

Supplemental Table S2: Senescence markers

Under normoxic conditions the expression of P16, P21 and SA β GAL was non-significant but there was a trend decrease in HC MSCs SA β GAL activity after treatment with hypoxia.

Supplemental Table S3: PCR markers

At baseline normoxic conditions, there was no difference in TGF -β,PGE, iONS, GAPDH, IDO,IL10, IL4 among the groups.

Supplemental Table S4A:
Correlation between MSC Function (normoxia) and Age, MSC Function (hypoxia) and Age

At the baseline and in hypoxic conditions, there was no correlation found between the function of MSCs and the age of the patients.

NORMOXIA

A.

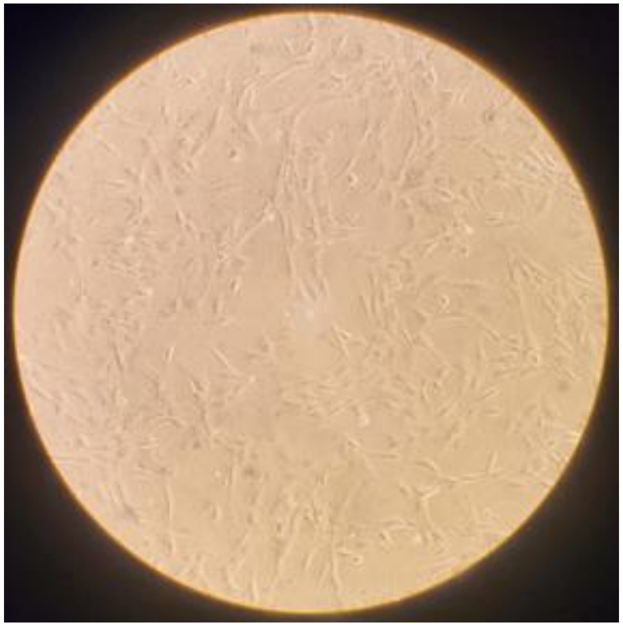

Low Resolution
4x objective

HYPOXIA

B.

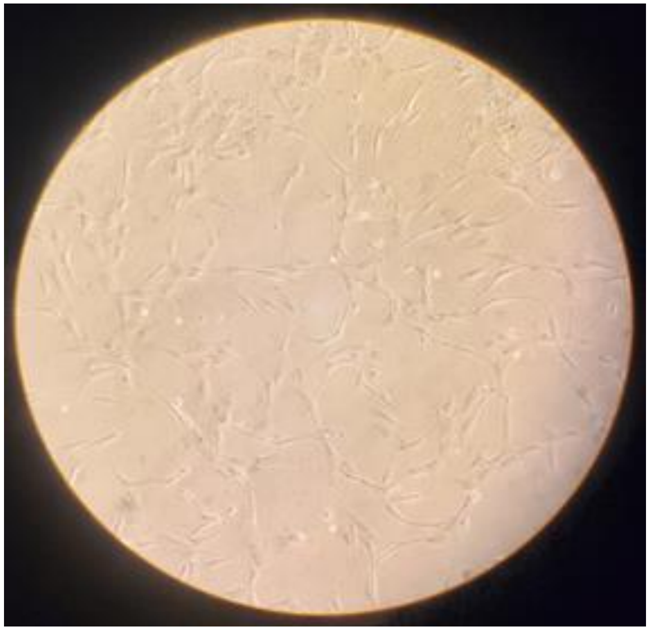

Low Resolution
4x objective

Fig. S1. Spindle-shaped morphology of cells isolated from adipose tissue of humans, characteristic of mesenchymal stem cells.

A. AMSC grown to confluence in Normoxia (20% O_2)
B. Cells grown to confluence and subjected to 48 hours of HPC (1% O_2)

AMSC, Adipose tissue derived mesenchymal stem cells; HPC, Hypoxia preconditioning, O_2, Oxygen

A.

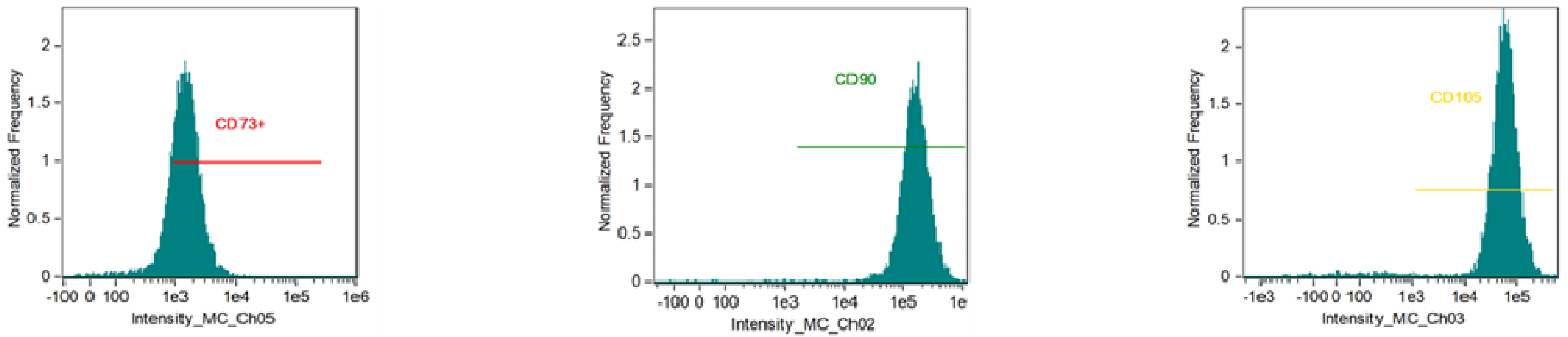

Intensity_MC_Ch05

Population	Count	%Gated
Single	10200	100
CD73+ & Single	8282	81.2

Intensity_MC_Ch02

Population	Count	%Gated
Single	10200	100
CD90 & Single	10180	99.8

Intensity_MC_Ch03

Population	Count	%Gated
Single	10200	100
CD105 & Single	10007	98.1

B.

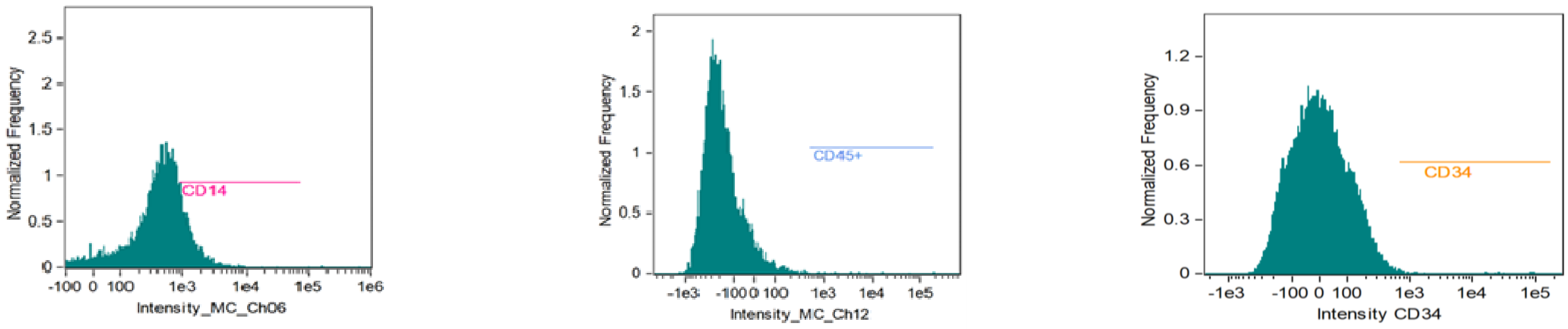

Intensity_MC_Ch06

Population	Count	%Gated
Single	9729	100
CD14 & Single	1712	17.6

Intensity_MC_Ch12

Population	Count	%Gated
Single	9729	100
CD45+ & Single	12	0.12

Intensity CD34

Population	Count	%Gated
Single	37798	100
CD34 & Single	111	0.29

Fig. S2. Flowcytometry characterizing the cell surface markers confirming that isolated cells were adipose tissue derived mesenchymal stem cells in Normoxia conditions.

A. **Cells strongly positive for markers CD73, CD90 and CD105**
B. **Cells negative for markers CD14, CD34 and CD45**

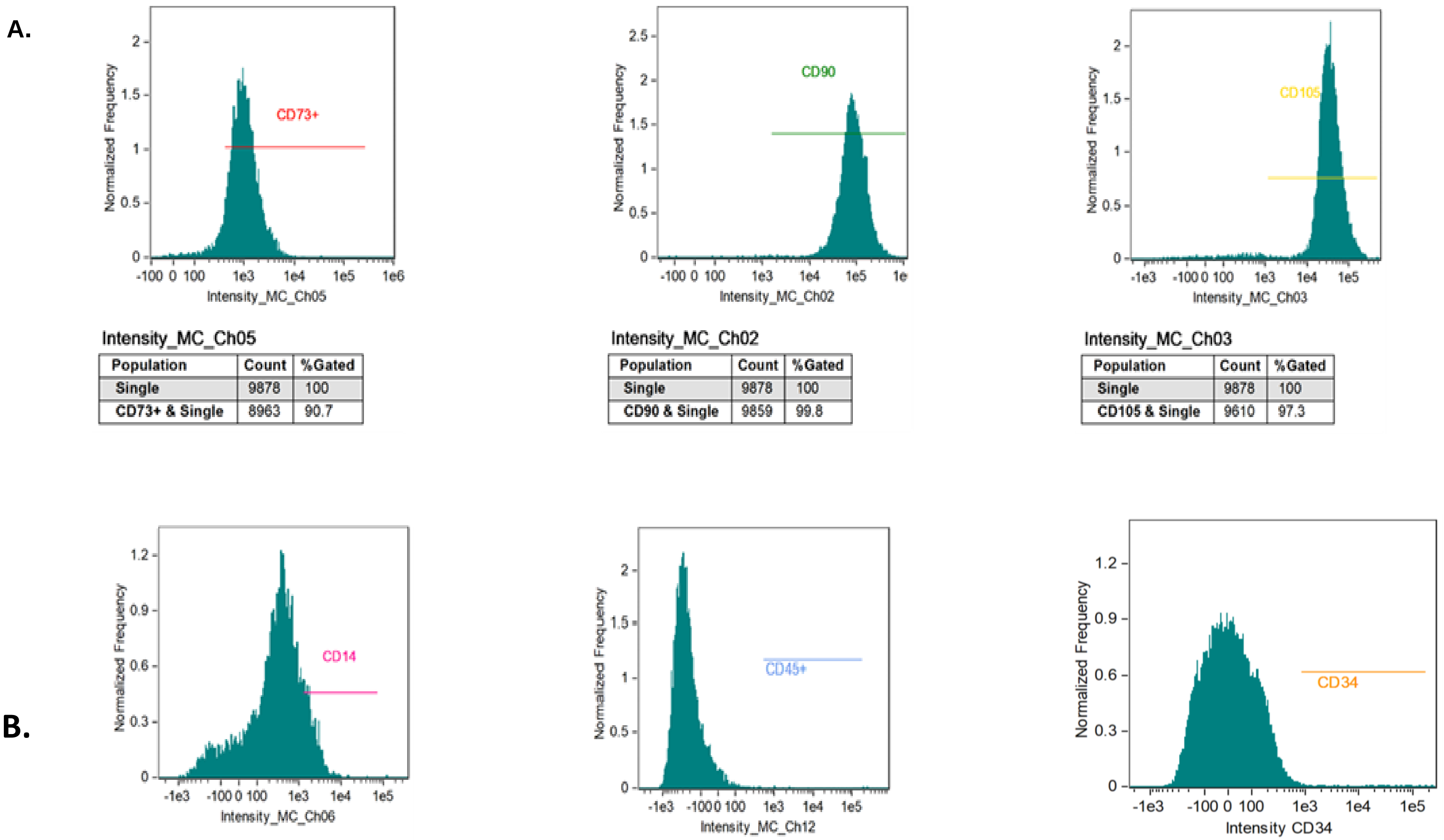

Intensity_MC_Ch05

Population	Count	%Gated
Single	9878	100
CD73+ & Single	8963	90.7

Intensity_MC_Ch02

Population	Count	%Gated
Single	9878	100
CD90 & Single	9859	99.8

Intensity_MC_Ch03

Population	Count	%Gated
Single	9878	100
CD105 & Single	9610	97.3

Intensity_MC_Ch06

Population	Count	%Gated
Single	9463	100
CD14 & Single	1126	11.9

Intensity_MC_Ch12

Population	Count	%Gated
Single	9463	100
CD45+ & Single	8	0.08

Intensity CD34

Population	Count	%Gated
Single	28559	100
CD34 & Single	60	0.21

Fig. S3. Flowcytometry characterizing the cell surface markers confirming that isolated cells were adipose tissue derived mesenchymal stem cells in Hypoxia conditions.

A. **Cells strongly positive for markers CD73, CD90 and CD105**
B. **Cells negative for markers CD14, CD34 and CD45**

A.

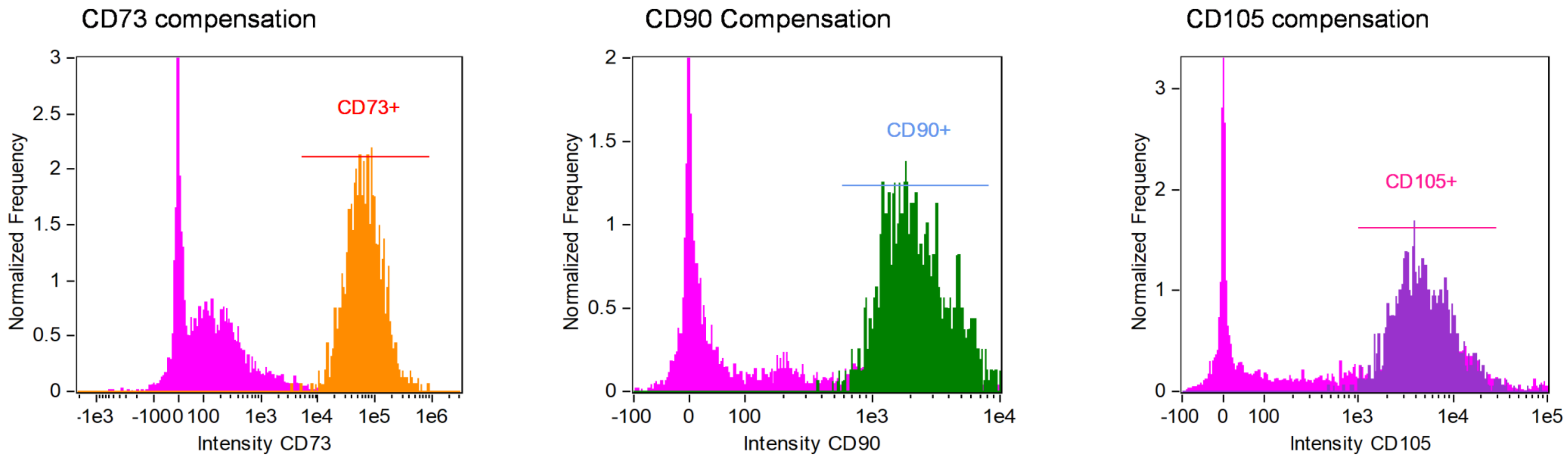

Fig. S4. A. Adipose tissue derived mesenchymal stem cells were stained with fluorescently conjugated antibodies against CD73, CD90, CD105 and the expression was analyzed by flow cytometry. The histogram in pink represents the isotype control and the histogram in orange, green, puple respectively represents the stained sample. Cells strongly positive for markers CD73, CD90 and CD105

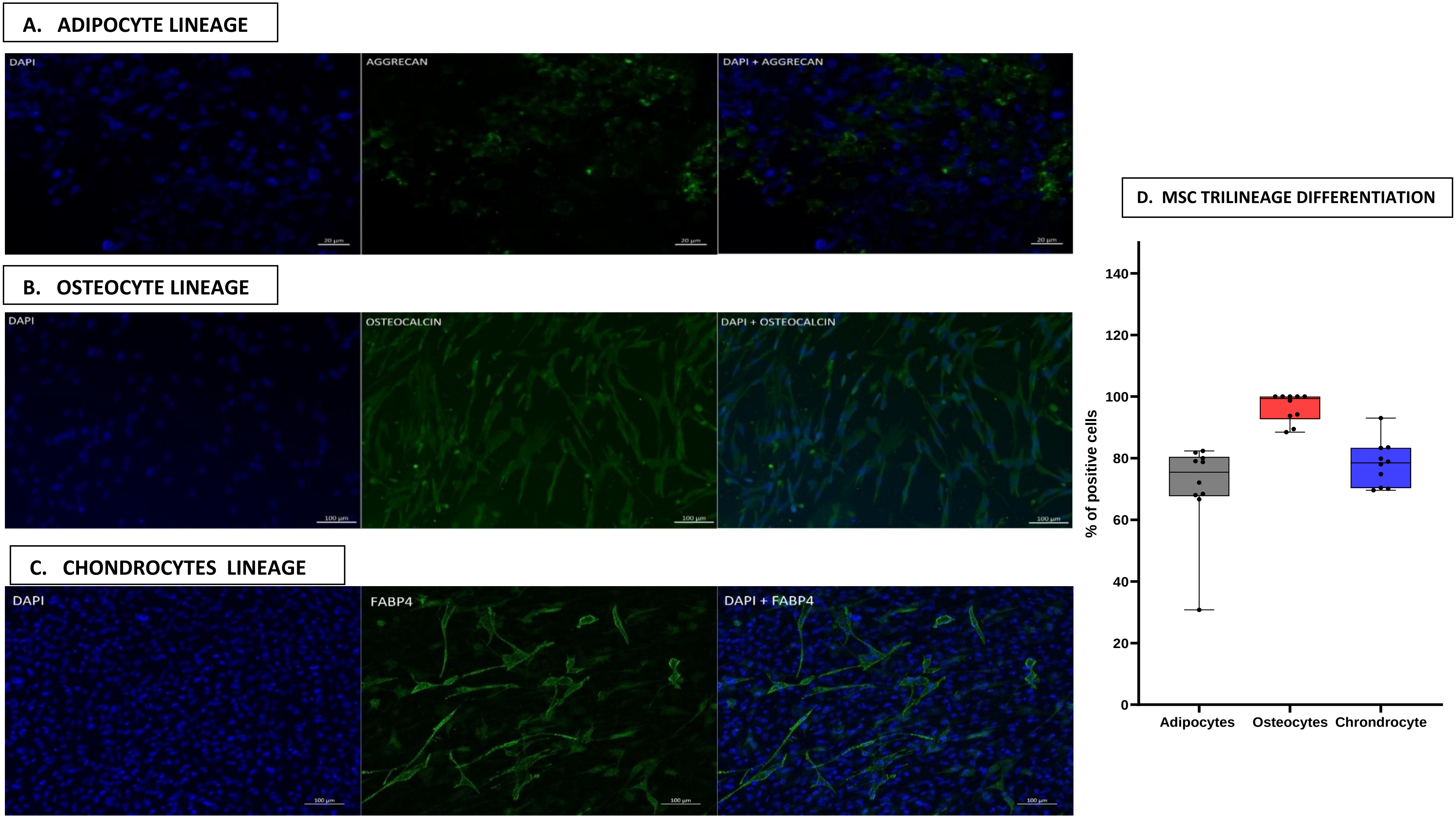

Fig. S5. Differentiation of cells grown after isolation from adipose tissue into adipocytes (A), osteocytes (B), chondrocytes (C), and quantification of adipocytes, osteocytes, and chondrocytes (D) quantification confirming their mesenchymal origin and regenerative properties. Images captured after fluorescent staining of differentiated cells.

Figure: S6

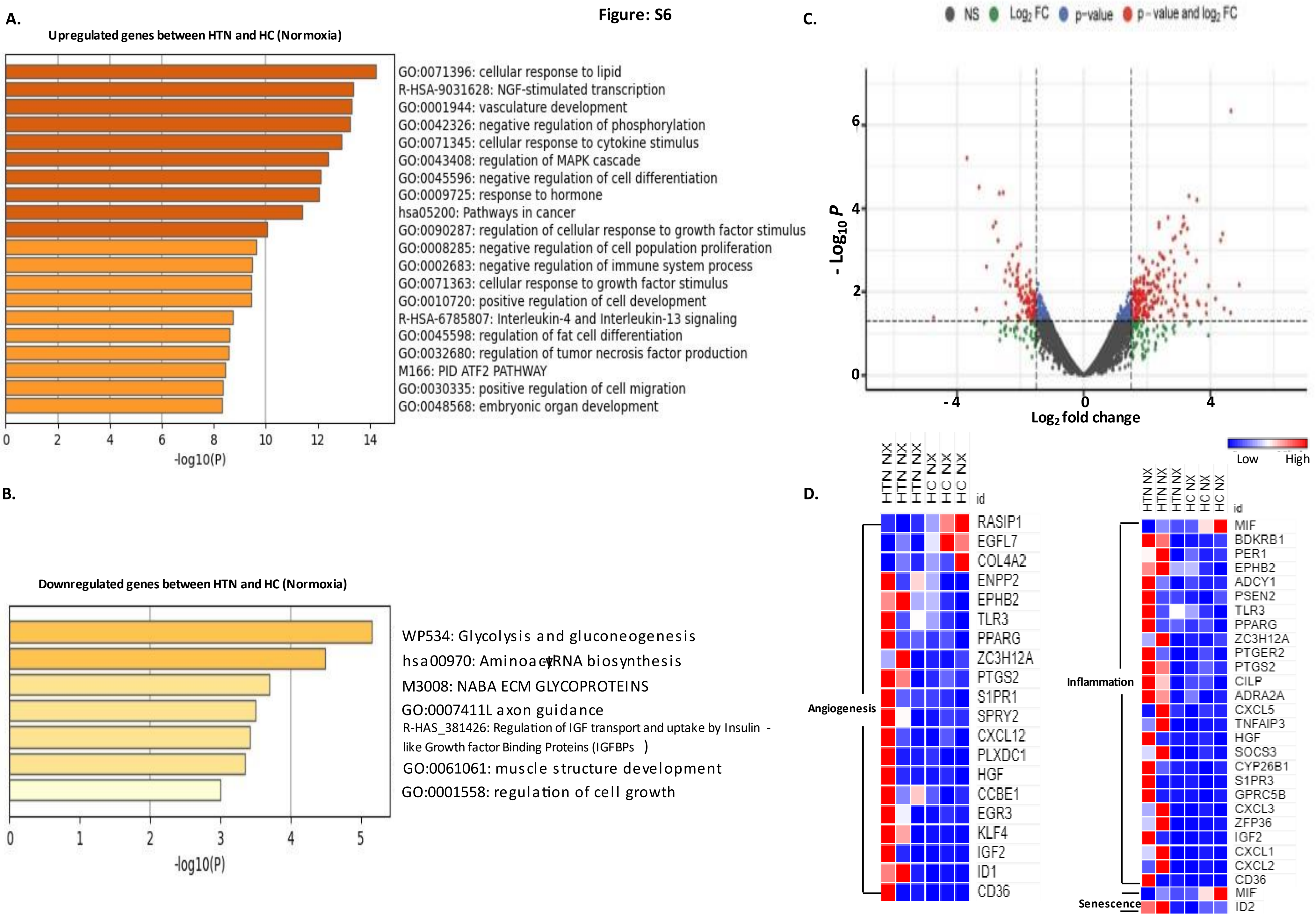
A.
Upregulated genes between HTN and HC (Normoxia)
GO:0071396: cellular response to lipid
R-HSA-9031628: NGF-stimulated transcription
GO:0001944: vasculature development
GO:0042326: negative regulation of phosphorylation
GO:0071345: cellular response to cytokine stimulus
GO:0043408: regulation of MAPK cascade
GO:0045596: negative regulation of cell differentiation
GO:0009725: response to hormone
hsa05200: Pathways in cancer
GO:0090287: regulation of cellular response to growth factor stimulus
GO:0008285: negative regulation of cell population proliferation
GO:0002683: negative regulation of immune system process
GO:0071363: cellular response to growth factor stimulus
GO:0010720: positive regulation of cell development
R-HSA-6785807: Interleukin-4 and Interleukin-13 signaling
GO:0045598: regulation of fat cell differentiation
GO:0032680: regulation of tumor necrosis factor production
M166: PID ATF2 PATHWAY
GO:0030335: positive regulation of cell migration
GO:0048568: embryonic organ development
0
2
4
6
8
10
12
14
-log10(P)
B.
Downregulated genes between HTN and HC (Normoxia)
WP534: Glycolysis and gluconeogenesis
hsa00970: Aminoacyl-tRNA biosynthesis
M3008: NABA ECM GLYCOPROTEINS
GO:0007411L axon guidance
R-HAS_381426: Regulation of IGF transport and uptake by Insulin - like Growth factor Binding Proteins (IGFBPs)
GO:0061061: muscle structure development
GO:0001558: regulation of cell growth
0
1
2
3
4
5
-log10(P)
C.
NS
Log2 FC
p-value
p - value and log2 FC
- Log10 P
6
4
2
0
- 4
0
4
Log2 fold change
D.
HTN NX
HTN NX
HTN NX
HC NX
HC NX
HC NX
id
RASIP1
EGFL7
COL4A2
ENPP2
EPHB2
TLR3
PPARG
ZC3H12A
PTGS2
S1PR1
SPRY2
CXCL12
PLXDC1
HGF
CCBE1
EGR3
KLF4
IGF2
ID1
CD36
Angiogenesis
Low
High
HTN NX
HTN NX
HTN NX
HC NX
HC NX
HC NX
id
MIF
BDKRB1
PER1
EPHB2
ADCY1
PSEN2
TLR3
PPARG
ZC3H12A
PTGER2
PTGS2
CILP
ADRA2A
CXCL5
TNFAIP3
HGF
SOCS3
CYP26B1
S1PR3
GPRC5B
CXCL3
ZFP36
IGF2
CXCL1
CXCL2
CD36
MIF
ID2
Inflammation
Senescence

Figure: S7

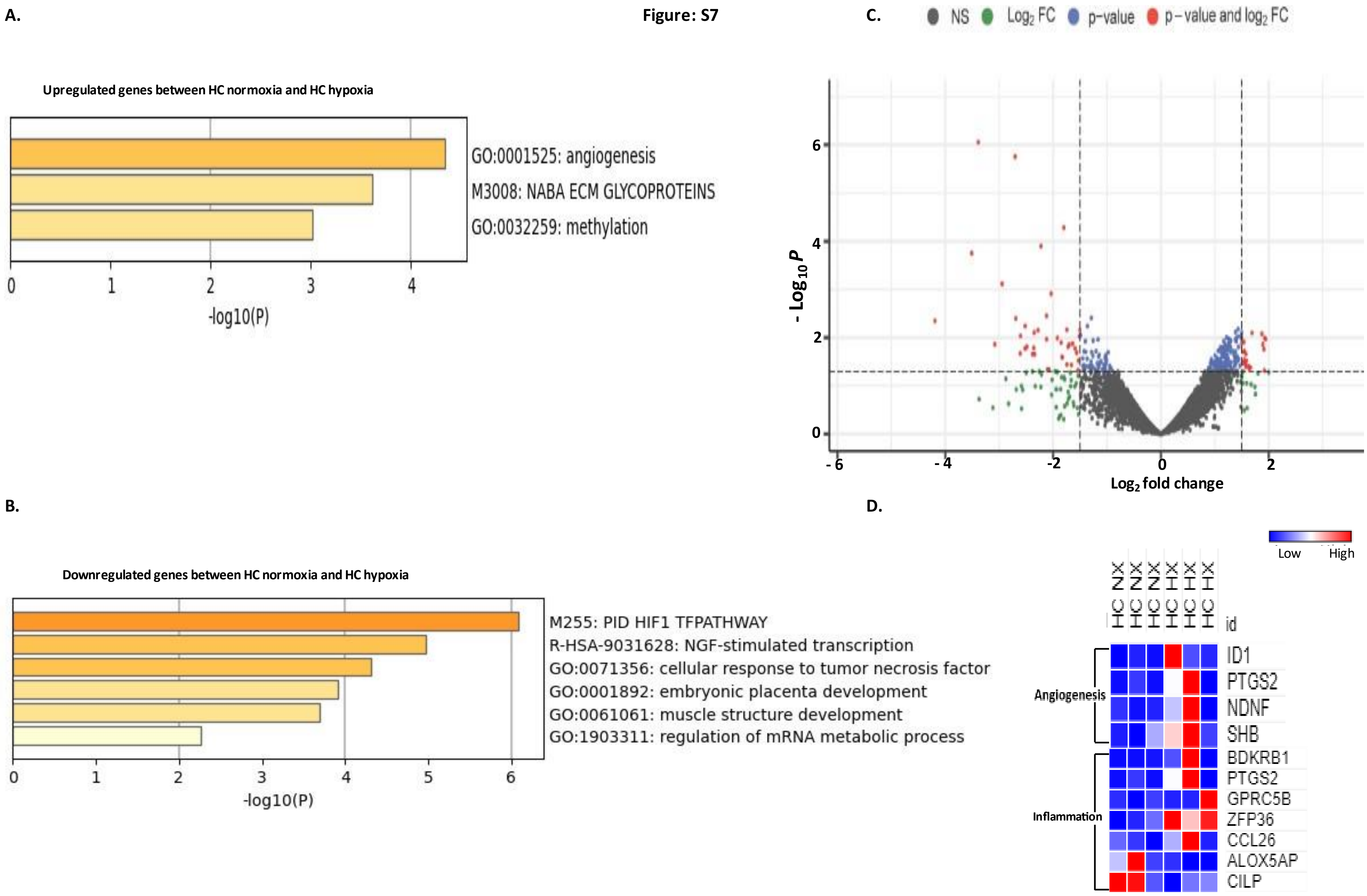

A.
Upregulated genes between HC normoxia and HC hypoxia
GO:0001525: angiogenesis
M3008: NABA ECM GLYCOPROTEINS
GO:0032259: methylation
0 1 2 3 4
-log10(P)
B.
Downregulated genes between HC normoxia and HC hypoxia
M255: PID HIF1 TFPATHWAY
R-HSA-9031628: NGF-stimulated transcription
GO:0071356: cellular response to tumor necrosis factor
GO:0001892: embryonic placenta development
GO:0061061: muscle structure development
GO:1903311: regulation of mRNA metabolic process
0 1 2 3 4 5 6
-log10(P)
C.
NS
Log2 FC
p−value
p−value and log2 FC
- Log10 P
0 2 4 6
- 6 - 4 -2 0 2
Log2 fold change
D.
Low
High
id
Angiogenesis
Inflammation
ID1
PTGS2
NDNF
SHB
BDKRB1
PTGS2
GPRC5B
ZFP36
CCL26
ALOX5AP
CILP

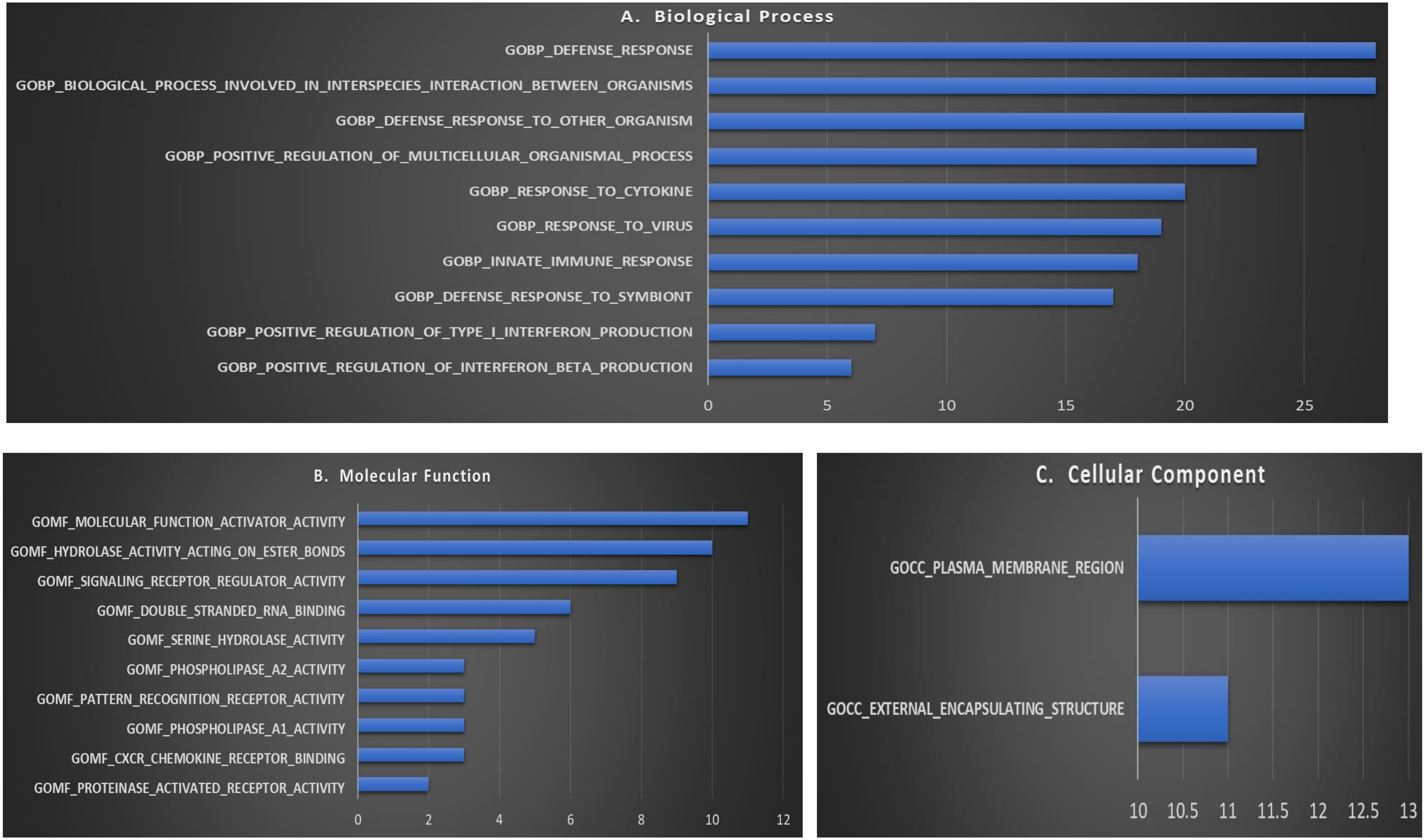

Fig.S8. Hypoxia HKD vs Normoxia HKD MSCs. Gene ontology (GO) analysis pathways (A) Biological Process (B) Molecular Functions (C) Cellular Components of Upregulated gene sets with significant changes between Hypoxia HKD and Normoxia HKD MSCs.

Figure: S9

Gene and transcription factors network interactions between HKD and HC (Normoxia)

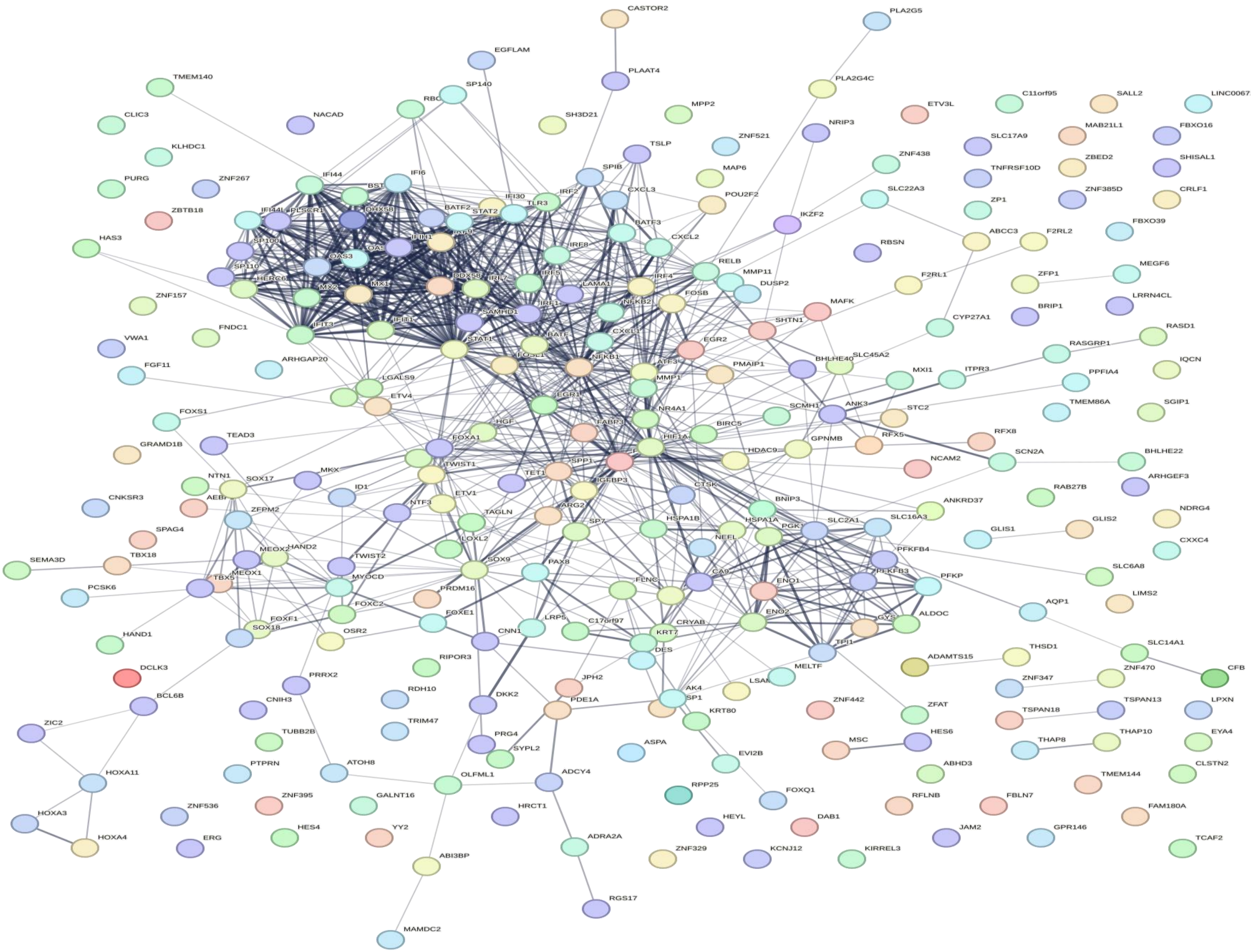

Figure: S10

Gene and transcription factors network interactions between HKD and HTN (Normoxia)

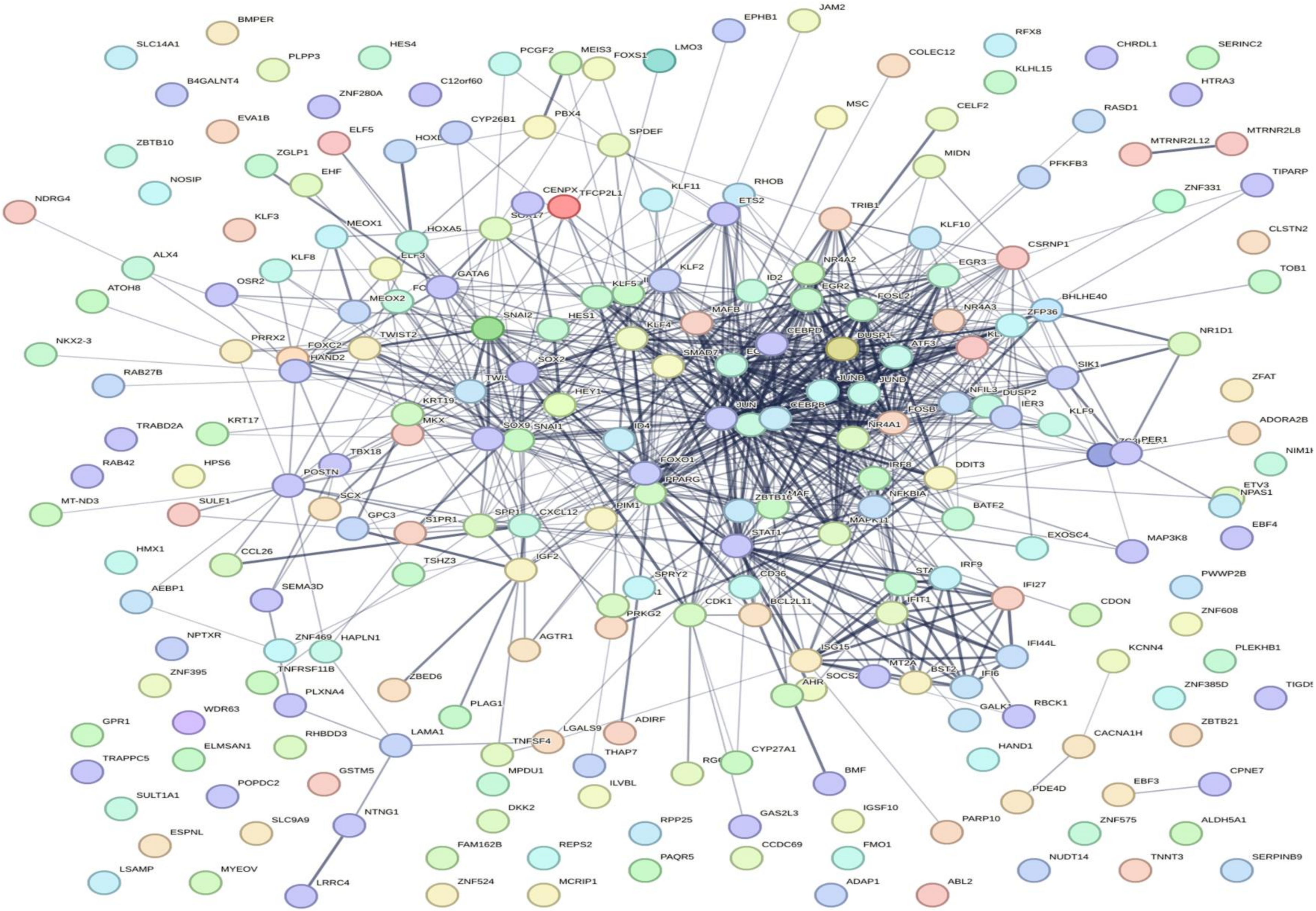

Figure: S11

Gene and transcription factors network interactions between HKD Normoxia and HKD Hypoxia

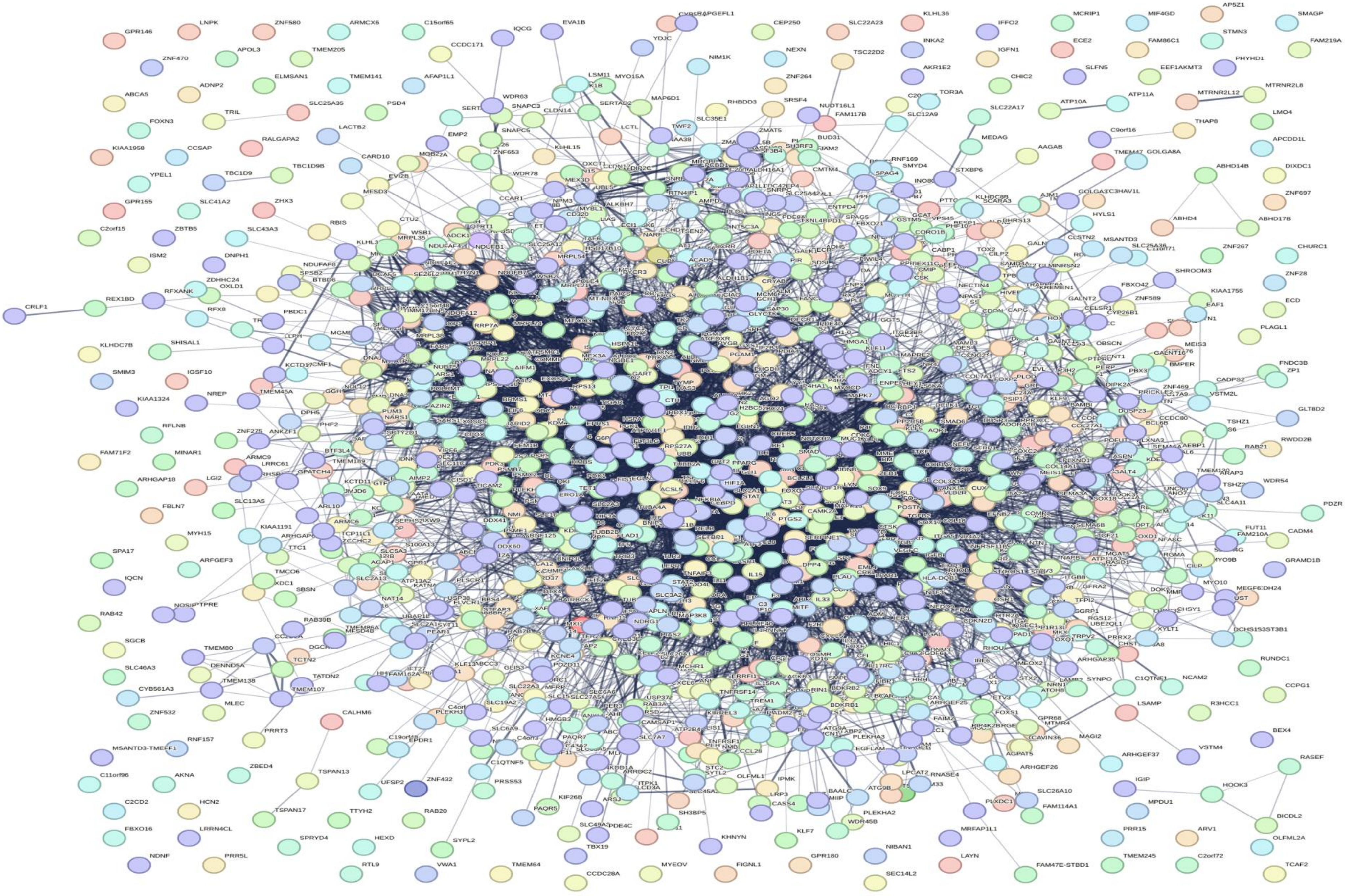

Figure S12

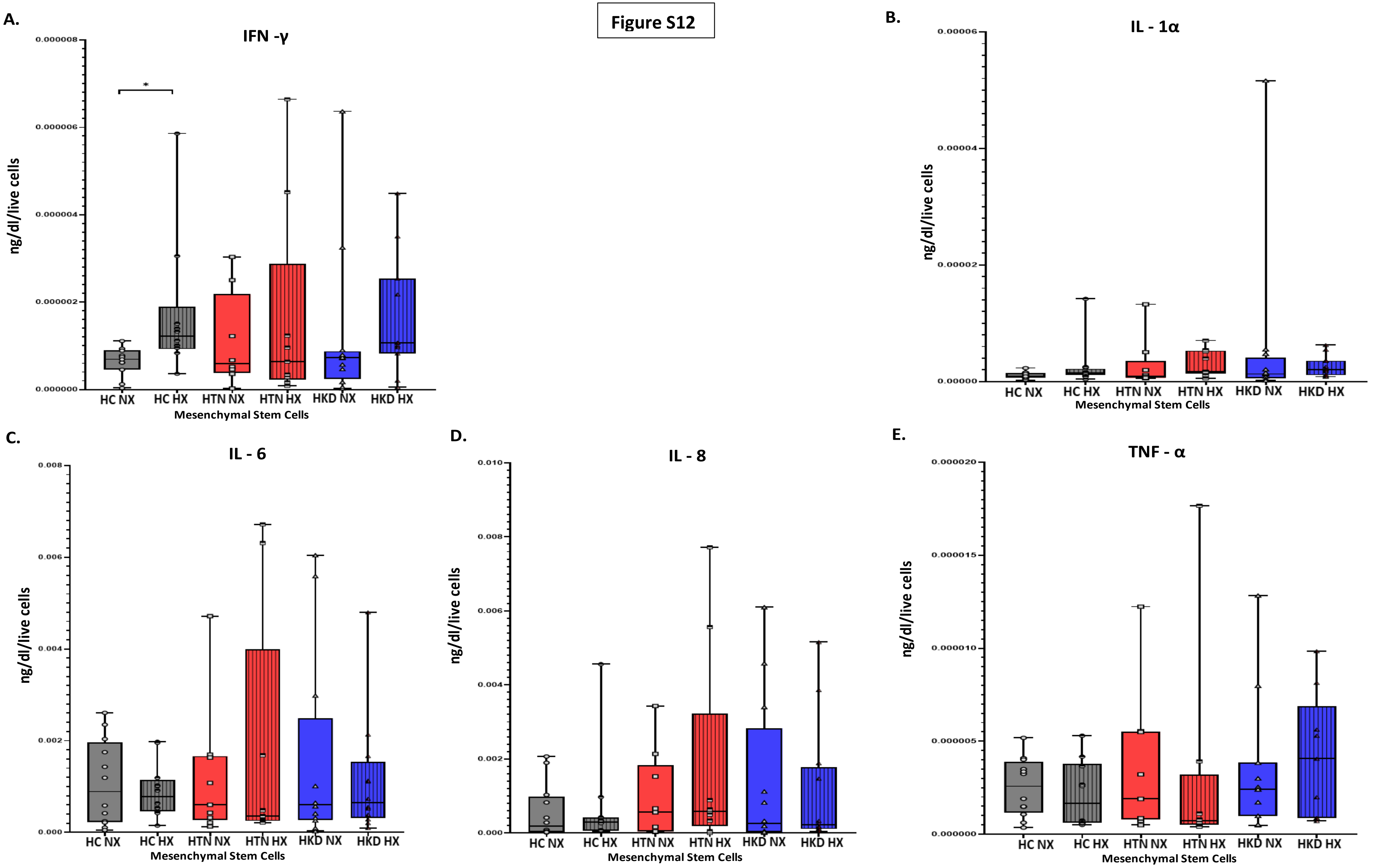
A.
IFN -γ
ng/dl/live cells
0.000008
0.000006
0.000004
0.000002
0.000000
*
HC NX
HC HX
HTN NX
HTN HX
HKD NX
HKD HX
Mesenchymal Stem Cells
B.
IL - 1α
0.00006
0.00004
0.00002
0.00000
C.
IL - 6
0.008
0.006
0.004
0.002
0.000
D.
IL - 8
0.010
0.008
0.006
0.004
0.002
0.000
E.
TNF - α
0.000020
0.000015
0.000010
0.000005
0.000000

Table S1- Angiogenesis secretome markers at baseline normoxia

VARIABLES	HC (12)	HTN (9)	HKD (12)	P value
EGF	$7.94108e^{-9}$ ($5.97554e^{-9}$ - $1.00493e^{-8}$)	$4.98339e^{-9}$ ($3.44828e^{-9}$- $1.5619e^{-8}$)	$7.19277e^{-9}$ ($5.9043e^{-9}$ - $1.10211e^{-8}$)	NS
HGF	$7.0609e^{-8}$($3.90828e^{-8}$- $2.02851e^{-7}$)	$7.03846e^{-8}$ ($3.43478e^{-8}$- $2.77636e^{-7}$)	$3.7796e^{-8}$ ($2.8515e^{-8}$ -$2.111e^{-7}$)#	NS
VEGF	0.00018 ($5.88315e^{-5}$- 0.000262995)	0.000338342 ($8.01248e^{-5}$- 0.001057009)	0.000119132 ($4.22891e^{-5}$- 0.000373018)	NS
VEGF (at gene level)	0.977101 (0.55706725- 1.3536075)	1.07 (0.64-1.435)	1.21 (0.79-1.46)	NS
EGF (at gene level)	0.97 (0.705- 2.1325)	3.06 (0.24- 5.68)	1.344779401 (0.425334961 - 3.110220509)	NS

Abbreviations: HC: healthy control; HTN: hypertension; HKD: Hypertensive kidney disease

Table S2- Senescence markers at baseline normoxia

VARIABLES	HC (12)	HTN (9)	HKD (12)	P value
P16	1.063 (0.430- 1.696)	1.160 (0.570- 1.480)	0.866 (0.472- 2.445)	NS
P21	0.810 (0.623- 1.190)	0.870 (0.610- 1.490)	0.895 (0.640- 1.113)	NS
SA β GAL	15765.5 (10393.500- 29550.500)	35322.5 (25168.500- 52886.000)	14956.5 (6672.875- 19085.000)	*0.04

Abbreviations: HC: healthy control; HTN: hypertension; HKD: Hypertensive kidney disease

Table S3 – PCR Markers at baseline normoxia

VARIABLES	HC (12)	HTN (9)	HKD (12)	P value
TGF -β	24.214 (23.488-25.148)	24.800 (24.441, 25.324)	25.182 (24.504, 25.484)	NS
PGE	26.827 (26.414- 27.859)	27.621 (26.550- 28.666)	27.020 (26.817- 27.341)	NS
iNOS	36.454 (35.355- 37.295)	38.396 (37.897- 39.677)	36.625 (36.351- 37.391)	NS
IDO	36.603 (34.912- 39.251)	38.831 (36.902- 39.447)	37.380 (35.873- 38.867)	NS
IL 10	38.702 (35.725- 39.716)	35.699 (34.445- 39.221)	36.374 (34.541- 38.427)	NS
IL 4	39.178 (37.917- 40.000)	39.067 (37.522- 40.000)	38.511 (36.663- 40.000)	NS
GAPDH	20.156 (19.052- 21.981)	21.375 (20.167- 22.746)	20.847 (20.310- 21.231)	NS

Abbreviations: HC: healthy control; HTN: hypertension; HKD: Hypertensive kidney disease; NS: non-significant

Table S4- Correlation between age and MSC function in hypoxic and normoxic conditions

	**Age**	
**MSC function**	**r**	**P**
NX Proliferation	0.16	0.36
HX Proliferation	0.17	0.33

Abbreviations: MSC: mesenchymal stem cell; NX: normoxia; HX: hypoxic

REFERENCE

[1] Isik B, Thaler R, Goksu BB, Conley SM, Al-Khafaji H, Mohan A, Afarideh M, Abumoawad AM, Zhu XY, Krier JD, Saadiq IM, Tang H, Eirin A, Hickson LJ, van Wijnen AJ, Textor SC, Lerman LO, Herrmann SM: Hypoxic preconditioning induces epigenetic changes and modifies swine mesenchymal stem cell angiogenesis and senescence in experimental atherosclerotic renal artery stenosis. Stem Cell Res Ther 2021, 12:240.
